# Ergothioneine, a dietary antioxidant improves amyloid beta clearance in the neuroretina of a mouse model of Alzheimer’s disease

**DOI:** 10.3389/fnins.2023.1107436

**Published:** 2023-03-14

**Authors:** Printha Wijesinghe, Clayton A. Whitmore, Matthew Campbell, Charles Li, Miranda Tsuyuki, Eleanor To, Justin Haynes, Wellington Pham, Joanne A. Matsubara

**Affiliations:** ^1^Department of Ophthalmology and Visual Sciences, Faculty of Medicine, Eye Care Centre, The University of British Columbia, Vancouver, BC, Canada; ^2^Department of Radiology and Radiological Sciences, Vanderbilt University Medical Center, Nashville, TN, United States; ^3^Vanderbilt University Institute of Imaging Science, Vanderbilt University Medical Center, Nashville, TN, United States; ^4^Djavad Mowafaghian Centre for Brain Health, The University of British Columbia, Vancouver, BC, Canada

**Keywords:** ergothioneine, Alzheimer’s disease, neuroretina, amyloid beta (Aβ), microglia, astrocytes, Aβ clearance

## Abstract

**Introduction:**

Ergothioneine (Ergo) is a naturally occurring dietary antioxidant. Ergo uptake is dependent on the transporter, organic cation transporter novel-type 1 (OCTN1) distribution. OCTN1 is highly expressed in blood cells (myeloid lineage cells), brain and ocular tissues that are likely predisposed to oxidative stress. Ergo may protect the brain and eye against oxidative damage and inflammation, however, the underlying mechanism remains unclear. Amyloid beta (Aβ) clearance is a complex process mediated by various systems and cell types including vascular transport across the blood–brain barrier, glymphatic drainage, and engulfment and degradation by resident microglia and infiltrating innate immune cells. Impaired Aβ clearance is a major cause for Alzheimer’s disease (AD). Here we investigated neuroretinas to explore the neuroprotective effect of Ergo in a transgenic AD mouse model.

**Methods:**

Age-matched groups of Ergo-treated 5XFAD, non-treated 5XFAD, and C57BL/6J wildtype (WT controls) were used to assess Ergo transporter OCTN1 expression and Aβ load along with microglia/macrophage (IBA1) and astrocyte (GFAP) markers in wholemount neuroretinas (*n* = 26) and eye cross-sections (*n* = 18). Immunoreactivity was quantified by fluorescence or by semi-quantitative assessments.

**Results and discussion:**

OCTN1 immunoreactivity was significantly low in the eye cross-sections of Ergo-treated and non-treated 5XFAD vs. WT controls. Strong Aβ labeling, detected in the superficial layers in the wholemounts of Ergo-treated 5XFAD vs. non-treated 5XFAD reflects the existence of an effective Aβ clearance system. This was supported by imaging of cross-sections where Aβ immunoreactivity was significantly low in the neuroretina of Ergo-treated 5XFAD vs. non-treated 5XFAD. Moreover, semi-quantitative analysis in wholemounts identified a significantly reduced number of large Aβ deposits or plaques, and a significantly increased number of IBA1(+)ve blood-derived phagocytic macrophages in Ergo-treated 5XFAD vs. non-treated 5XFAD. In sum, enhanced Aβ clearance in Ergo-treated 5XFAD suggests that Ergo uptake may promote Aβ clearance possibly by blood-derived phagocytic macrophages and *via* perivascular drainage.

## 1. Introduction

Alzheimer’s disease (AD) is the most prevalent form of dementia and accounts for 60–80% of all dementia (Duong et al., 2017). Accumulating evidence highlights that a deficit in the clearance of amyloid beta (Aβ) protein, which leads to the formation of insoluble Aβ plaques, plays a major role in the pathogenesis of AD. Clearance of cerebral Aβ is a complex process mediated by various systems and cell types, including vascular transport across the blood–brain barrier, glymphatic drainage, and engulfment and degradation by resident microglia and infiltrating innate immune cells (Tarasoff-Conway et al., 2015; Zuroff et al., 2017). Each of the above systems and cell types likely contributes to Aβ clearance to varying degrees; however, their cumulative effects are essential for Aβ homeostasis. Thus, defects in any singular process may cause or predispose the brain to pathologic Aβ accumulation, and consequently development of AD (Zuroff et al., 2017).

Microglia are considered as the resident macrophages of the central nervous system (CNS) and serve as a first line of defense against pathogens and injury in the brain (Glass et al., 2010; Wyss-Coray and Rogers, 2012). In AD brains, histological evidence has indicated that activated microglia surround senile plaques, along with reactive astrocytes, as well as these cells stain positive for inflammatory markers (Akiyama et al., 2000; Glass et al., 2010). Reduced Aβ uptake capacity of microglia in the brain has been suggested as a major cause for AD (Lee and Landreth, 2010; Krabbe et al., 2013). The role of microglia in Aβ clearance however remains inconclusive. Some studies have shown that microglia are poor phagocytes of Aβ and thus cannot play a significant role in Aβ clearance or plaque remodeling, neither promoting or protecting against Aβ induced pathology (Grathwohl et al., 2009; Gate et al., 2010). Whereas some other studies indicated that microglial cells are capable of remodeling and enhancing the clearance of Aβ plaques (Kiyota et al., 2009; Chakrabarty et al., 2010; Gate et al., 2010). Other than microglia, growing evidence indicate that astrocytes can be phagocytic, involved in the elimination of debris and synaptic remodeling (Morizawa et al., 2017). Recently, Konishi et al. (2020) demonstrated that astrocytic phagocytosis is a compensatory mechanism for microglial dysfunction for the maintenance or prolongation of a healthy CNS. Konishi et al.’s (2020) study suggests that activated astrocytes engulf microglial debris as CNS-associated macrophages and circulatory inflammatory monocytes did not clear microglial debris. Furthermore, a recently discovered glymphatic pathway in which astrocytes allow the movement of fluid between paravascular spaces and the interstitium *via* water channels [more specifically aquaporin 4 (AQP4)] appears to be important for Aβ clearance (Iliff et al., 2013).

Besides glial cells, blood immune cells have emerged as more effective at neuroprotection, neuroinflammation regulation and Aβ clearance than microglia in AD (Simard et al., 2006; Butovsky et al., 2007; Hawkes and McLaurin, 2009; Koronyo-Hamaoui et al., 2009; Koronyo et al., 2015). These studies suggested that Aβ generated in the brain diffuse into the blood possibly by infiltrating monocytes and be cleared in the periphery, implying that the peripheral blood system also plays an essential role in clearing Aβ from the brain. In addition, some clinical investigations have shown that peripheral monocytes isolated from AD patients seem ineffective at phagocytosing Aβ and may even contribute to cerebral amyloid angiopathy (Fiala et al., 2005; Malm et al., 2005; Zaghi et al., 2009; Mildner et al., 2011). Therefore, it is possible that microglia and/or blood immune cells become dysfunctional and lose their ability to degrade Aβ in the pathogenesis of AD.

Ergothioneine (Ergo), a naturally occurring amino acid with considerable *in vitro* antioxidant properties (Ey et al., 2007), is abundantly found in mushroom which is a major source for Ergo in the human diet. The organic cation transporter novel-type 1 (OCTN1) is responsible for Ergo uptake and accumulation in human tissues from the diet and the selective distribution to tissues owing to differential OCTN1 expression (Gründemann et al., 2005; Gründemann, 2012; Tang et al., 2018; Engelhart et al., 2020). High expression of OCTN1 and therefore high levels of Ergo is observed in cells and tissues such as myeloid lineage cells (granulocytes and monocytes), bone marrow, ocular tissues and brain tissues that are likely predisposed to oxidative stress (Uhlén et al., 2015; Tang et al., 2018). Ergo may protect the brain against oxidative damage and neuroinflammation, target underlying pathologies in neurodegeneration such as mitochondrial dysfunction and toxic amyloid accumulation, and even promote neurogenesis (Cheah and Halliwell, 2021). In human brain microvascular endothelial cells, Ergo exhibited vascular protection (Li et al., 2017) and the expression of OCTN1 in cerebral endothelial cells is suggested as a crucial mechanism that facilitates neuroprotective substrates to access brain parenchyma. Ishimoto et al.’s (2018) study demonstrated the functional expression of OCTN1 in murine cultured microglial cells and suggested that OCTN1 expression negatively regulates the induction of inflammatory cytokine interleukin-1β. Moreover, clinical studies have shown that lower plasma levels of Ergo is associated with the incidence of several disorders including mild cognitive impairment (Cheah et al., 2016) and Parkinson’s disease (Hatano et al., 2014), suggesting the perception that a deficiency in Ergo may elevate the risk of disease.

As the retina and brain are derived from the same tissues during embryonic development, it has been suggested that the retina is a window to the brain, in that, cellular processes that occur in the brain also occur in the retina. Several proposed mechanisms have been suggested for the changes in the AD retina such as neurodegeneration, proinflammation, amyloid misfolding, and amyloid angiopathy (Ning et al., 2008; Koronyo et al., 2017; Lee et al., 2020; Sidiqi et al., 2020). Further, retina is one of the highest oxygen-consuming tissues in the human body (Schmidt et al., 2003). Therefore, in this study, we investigate the neuroprotective effect of Ergo in the retinas of a transgenic AD mice. Here we propose that the increased level of Ergo intake, particularly by resident microglia and/or blood-derived macrophages, enhances Aβ clearance by phagocytosis and *via* perivascular clearance system.

## 2. Materials and methods

### 2.1. Animals

Transgenic 5XFAD mice obtained from Jackson Laboratories were maintained at Vanderbilt University Animal Care Unit under standard conditions, in a 12-h light/dark cycle and with free access to food and water. These animals were genotyped using tail or ear derived DNA to confirm the expression of genes, 377 bp amyloid precursor protein (*APP*) and 608 bp presenilin 1 (*PSEN1*) using polymerase chain reaction based 1% agarose gel electrophoresis. From these animals, nine Ergo-treated 5XFAD (4–5 months old, three females and six males), nine non-treated 5XFAD (4–5 months old, five females and four males), and eight non-treated C57BL/6J wildtype (WT controls) (4–5 months old, four females and four males) mice were used for the investigations. C57BL/6J is the background strain for 5XFAD transgenic mice. Therefore, in addition to Ergo-treated vs. non-treated 5XFAD, C57BL/6J wildtype was included as a control to determine the level of expression of Aβ (refer Supplementary File which describes 6E10 immunoreactivity in transgenic and WT mice) as well as other protein markers, and to explore the existing Aβ clearance mechanisms.

Commercially purchased (Cayman Chemical, Ann Arbor, MI, USA) L-Ergo, formulated in sterilized water was administrated orally without purifications steps as recommended by the manufacturer. Nine 5XFAD mice, starting at the age of 1.5 months, were subjected to oral gavage treatment with high doses of Ergo solution at a concentration of 50 mg/kg 3 times per week over the course of 8 weeks. For every gavage treatment, freshly prepared Ergo solution was used. This procedure involves passing a reusable oral gavage needle through the mouth and placing the rounded tip atop of the esophagus of an awake animal in a manner to encourage the animal to swallow the formulation voluntarily. The curvature of the needle fitting with oral cavity, along with the extra smooth round ball stainless steel tip ensured minimal discomfort to the treated animals. To ameliorate potential compounding stress-induced discrepancies, non-treated 5XFAD and WT controls similarly underwent oral gavage procedure with sterilized water. Animals were sacrificed at the age of 4–5 months for subsequent histopathological studies.

### 2.2. Cardiac perfusion and tissue collection

Cardiac perfusion was carried out under ice-cold conditions. A sharp incision was made into the abdomen of the anesthetized mouse, followed by a longitudinal cut with a scalpel to open the thoracic cavity, which then was stabilized with a retractor. Perfusion began by inserting a 20-gauge syringe containing ice-cold 1× phosphate-buffered saline (PBS) (30 ml, pH 7.4) in the left ventricle while the atrium was snipped off, and then followed by injection of 4% paraformaldehyde solution. Once the perfusion was completed, the animals were decapitated, eyes were harvested immediately. Whole eye globes were then fixed in 4% paraformaldehyde at 4°C for a period of 1–2 weeks. Right eye or oculus dexter (OD) was used for wholemounts, while left eye or oculus sinister (OS) was used for the cross-sectional studies.

### 2.3. Preparation of wholemount neuroretinas for confocal microscopy

Briefly, the cornea was removed by cutting in a circular path along with the ora serrata using small scissors while holding the eye at the limbus with forceps. Next, the lens and vitreous body were removed with blunt-edged forceps. The neuroretina was then carefully detached from the eyecup using fine-tipped sable brushes positioned between the neuroretina and the eyecup. The optic nerve was cut at the location between the neuroretina and the eyecup to ease the separation of the neuroretina from the eyecup. The neuroretina was then cut into half to make two hemi-retinal wholemounts, rinsed with PBS (pH 7.2–7.4) and the thin strands of vitreous body were removed under a dissection microscope.

### 2.4. Co-localization of Aβ protein with astrocyte or microglia/macrophage markers in the wholemount neuroretinas using double immunostaining techniques

For this work, nine Ergo-treated 5XFAD (4–5 months old, three females and six males), nine non-treated 5XFAD (4–5 months old, five females and four males), and eight WT controls (4–5 months old, four females and four males) were used. To determine Aβ burden, we used 6E10 primary antibody (mouse monoclonal, Cat# 803001, BioLegend) which is a marker for 1–16 amino acid residues of Aβ peptide (refer Supplementary File which describes 6E10 immunoreactivity in transgenic and WT mice). Other primary antibodies included glial fibrillary acidic protein (GFAP, rabbit poly clonal, Cat# Z0334, Dako), a type III intermediate filament protein and a marker for astrocytes and ionized calcium-binding adapter molecule 1 (IBA1, rabbit polyclonal, Cat# 019-19741, Wako), a marker for microglia/macrophage-specific calcium-binding protein. Secondary antibodies Alexa Fluor™ 546 goat anti-mouse IgG1 (Cat# 21123) and Alexa Fluor^®^ 488 goat anti-rabbit IgG (Cat# 11070) were used for fluorescence confocal microscopy.

In this work, one half of the neuroretina (i.e., hemi-retinal wholemount) was used for 6E10 and IBA1 double immunostaining and the other half was used for 6E10 and GFAP double immunostaining. Briefly, neuroretinas were placed carefully in 48-well plates containing PBS and rinsed 3 times, each for 5 min. Formic acid (88%) was then added to each well for antigen retrieval and incubated for 5 min at room temperature (RT). Samples were then washed in PBS. After the washing steps with PBS (3 times, each for 5 min), samples were incubated with blocking buffer (3% normal goat serum plus 0.3% Triton X-100 in PBS) for 20 min at RT. Primary antibodies 6E10 (1:100) and GFAP or IBA1 (1:100) diluted in 3% blocking buffer were then added to each well and incubated at RT for 1 h, followed by 72 h at 4°C with gentle agitation. Next, the wholemounts were washed with PBS (4 times, each for 5 min), and then secondary antibodies, Alexa Fluor™ 546 (1:500) and Alexa Fluor^®^ 488 (1:500) diluted in 1% blocking buffer were added to each well and incubated at RT for 45 min. Next, the wholemounts were washed with PBS (4 times, each for 15 min), and the nuclei were stained with 4′,6-diamidino-2-phenylindole dihydrochloride (DAPI) (1:500) for 10 min, followed by washing with PBS for 4 times, each 15 min. Finally, neuroretinas were mounted onto the glass slides using mounting medium containing 80% glycerol and 20% PBS, covered with #1.5 thickness coverslips for confocal microscopy. Negative control slides were simultaneously processed, but with the omission of primary antibodies and washed separately to avoid the cross contaminations throughout the remaining steps.

### 2.5. Localization of OCTN1 transporters and co-localization of 6E10 with GFAP or IBA1 or AQP4 markers in the eye cross-sections

For this work, six Ergo-treated 5XFAD (4–5 months old, two females and four males), six non-treated 5XFAD (4–5 months old, three females and three males), and six WT controls (4 months old, three females and three males) were used. In addition to 6E10, GFAP, and IBA1, primary antibodies OCTN1 (rabbit polyclonal, Cat# ACT-014, Alomone Labs), a marker for Ergo transporter and AQP4 (rabbit polyclonal, Cat# AQP-004, Alomone Labs), a major membrane water channel in the CNS were used in the eye cross-sections. Six micrometer thick eye cross-sections, after a series of deparaffinization and rehydration steps, underwent pre-treatment with 88% formic acid for 5 min at RT for double immunostaining with 6E10 and then rinsed in PBS. Antigen retrieval was carried out with 0.05% proteinase K in Tris-EDTA buffer (pH 8.0) for all 5 primary antibodies and incubated for 10 min at RT. Next, sections were incubated in blocking buffer (3% normal goat serum and 0.3% Triton X-100 in PBS) for 20 min at RT. Primary antibodies OCTN1 (1:200), 6E10 (1:200), GFAP (1:200), IBA1 (1:200), and AQP4 (1:100) diluted in 3% blocking buffer were then added to each section and incubated at RT for 1 h, followed by overnight at 4°C. Following washings with PBS, secondary antibodies, Alexa Fluor™ 546 (1:500) and Alexa Fluor^®^ 488 (1:500) diluted in PBS were added to each section and incubated at RT for 45 min. After washing steps, nuclei were stained with DAPI. Sections were finally mounted using 80% glycerol plus 20% PBS mounting medium with #1.5 thickness coverslips and stored at 4°C for confocal microscopy. Negative control slides were simultaneously processed, but with the omission of primary antibodies and washed separately to avoid the cross contaminations throughout the remaining steps.

### 2.6. Image analysis

All fluorescent images were captured using Zeiss LSM 800 confocal microscope with ZEN 2.6 (blue edition) software. Cy3 immunofluorescence from the 6E10 antibody was imaged at 543 nm, Alexa Fluor^®^ 488 immunofluorescence from the GFAP, IBA1, AQP4, and OCTN1 antibodies was imaged at 488 nm, and the nuclear labeling with DAPI was imaged at 405 nm for 200× (scale bar 50 μm) and 400× (scale bar 20 μm) magnifications. Confocal settings including laser, pinhole, and master gain were same across the three animal groups for each marker whereas digital gain and offset kept at default settings throughout the study. For the double labeled wholemounts (6E10 and GFAP or IBA1), a series of *z*-stack images (200×) were taken in an average of five non-overlapping fields focusing on the level of retinal ganglion cell layer (GCL). The GCL was identified by sequentially imaging through the thickness of the wholemount, starting from inner limiting membrane (ILM)/nerve fiber layer (NFL), then proceeding to the GCL (in which, DAPI labeled nuclei are abundant), inner plexiform layer (IPL, a cell free layer) and finally into the inner nuclear layer (INL, in which DAPI labeled nuclei are abundant). The series of *z*-stack images confirmed that the antibody incubations (72 h) penetrated into the thick wholemounts, as labeling was observed as deep as the INL of *z*-stack images. For the 6E10 and GFAP or IBA1 double labeled eye cross-sections, *z*-stack images (400×) were taken in a minimum of two central and two peripheral fields of the retina starting at the level of the ILM/NFL to outer nuclear layer (ONL). For the OCTN1 labeled eye cross-sections, *z*-stack images (400×) were taken from anterior segments of the eye such as cornea, lens and the ciliary body, and posterior segments of the eye such as central and peripheral retinas and retinal pigment epithelium (RPE)/Choroid regions. And, for the 6E10-AQP4 labeling, images were captured at 200× magnification in a minimum of four central and two peripheral retinal regions.

#### 2.6.1. Measurement of immunoreactivity using pixel counts

Orthogonal projections of *z*-stacks and single snap tiff files were used to calculate pixel counts using ImageJ software by a minimum of two independent investigators. Areas that displayed damaged or torn tissues and areas that displayed artifacts were excluded from the pixel count analysis. Across the three animal groups, immunoreactivity for each marker was measured at same confocal settings, quantified at same radius and threshold settings and presented per mm^2^. As the OCTN1 expression was quantified in detail including both posterior segments such as retina and RPE/Choroid, and anterior segments such as cornea, ciliary body, and lens of the eye, four animals per group were used for image analysis.

#### 2.6.2. Semi-quantification

Since the pixel counts obtained from the wholemounts reflected both Aβ depositions and Aβ clearance on the surface, a semi-quantification protocol was adapted to count the presence of insoluble 6E10+(ve) neuritic type Aβ plaques or large deposits manually. In addition, the number of IBA1+(ve) and GFAP+(ve) phagosome-like structures (PLS) were counted on the surface of wholemount neuroretinas. IBA1+(ve) PLS (resident or blood-derived macrophages) was identified by 6E10+(ve) Aβ deposits that were engulfed or surrounded by DAPI+(ve) blood-derived macrophages or multiple activated ameboid microglia as described by Zhao et al. (2015). GFAP+(ve) PLS (phagocytic astrocytes or green florescence positive phagosomes) was identified by 6E10+(ve) Aβ deposits that were engulfed or surrounded by DAPI+(ve) clumps of nuclei. Debris or autofluorescence bodies were distinguished from the functional PLS based on triple (6E10, GFAP/IBA1, and DAPI) staining and excluded. For this purpose, each *z*-stack (1 μm thick slices from ILM/NFL to INL) obtained at 200× magnification was screened manually by a minimum of two independent investigators across the three animal groups.

### 2.7. Statistical analysis

Due to the sex-based differences in the severity of AD pathology in 5XFAD mice, we initially compared 6E10 immunoreactivity between male and female findings within Ergo-treated and non-treated 5XFAD groups using independent samples *t*-test (Student’s or Welch’s corrected) for both wholemounts and cross-sections (central and peripheral retinas). However, sex-based analysis did not show any significant difference (*p* > 0.05) in 6E10 immunoreactivity between male and female mice within Ergo-treated and non-treated 5XFAD groups for wholemounts (*p* = 0.147 and *p* = 0.613, respectively), central retina (*p* = 0.566 and *p* = 0.49, respectively) and peripheral retina (*p* = 0.436 and *p* = 0.163, respectively). Therefore, pixel counts and semi-quantitative counts were compared across WT controls, non-treated 5XFAD, and Ergo-treated 5XFAD by combining male and female data. Pixel counts were analyzed using non-parametric one-way ANOVA test (Kruskal–Wallis H test, two-sided) followed by Dunn’s *post-hoc* test at a Bonferroni adjusted significance level of *p* < 0.05 for multiple comparisons. Semi-quantitative counts were analyzed using one-way ANOVA test followed by Games-Howell *post-hoc* test at a significance level of *p* < 0.05 for multiple comparisons. Spearman’s rank-order correlation test was conducted to see the association between 6E10 and other tested protein markers at a significance level of *p* < 0.05 based on the planes of section. SPSS version 25.0 (IBM Corp., Armonk, NY, USA) was used for the statistical analysis, and the GraphPad Prism 9 (GraphPad Software Inc., San Diego, CA, USA) was used to generate graphs.

## 3. Results

### 3.1. Localization of OCTN1 transporters in the eye cross-sections

**FIGURE 1:**
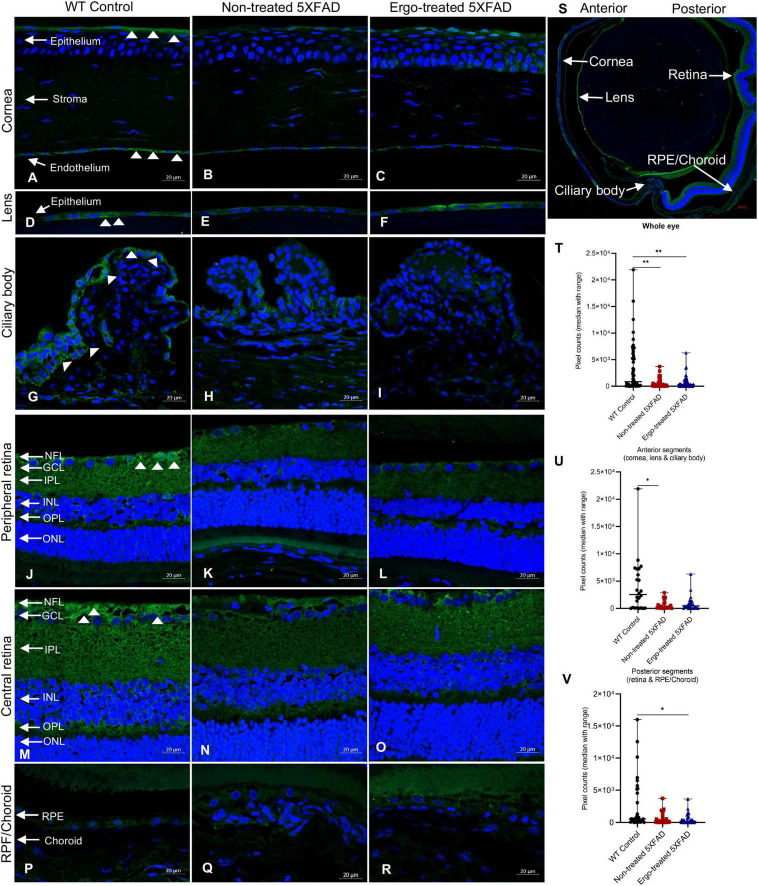
Localization of novel organic cation transporter 1 (OCTN1) in the eye cross-sections. OCTN1 immunoreactivity in anterior (cornea, lens, and ciliary body) and posterior (peripheral and central retina, RPE/Choroid) segments of the eyes of **(A,D,G,J,M,P)** WT controls, **(B,E,H,K,N,Q)** non-treated 5XFAD, and **(C,F,I,L,O,R)** Ergo-treated 5XFAD, respectively are illustrated. **(S)** OCTN1 labeled whole eye cross-section to illustrates the anterior and posterior segments that were used for pixel count analysis. OCTN1 immunoreactivity in the **(T)** whole eye, **(U)** anterior, and **(V)** posterior segments were compared across the three animal groups and illustrated using scatter plots. White arrowheads indicate OCTN1 labeling in the eye cross-sections. Significance levels of *p* < 0.05 are indicated as *p < 0.05 and ^**^p < 0.01. Scale bar, 20 μm; bright green, OCTN1; blue, DAPI. OCTN1, organic novel cation transporter 1; WT, C57BL/6J wildtype; NFL, nerve fiber layer; GCL, ganglion cell layer; IPL, inner plexiform layer; INL, inner nuclear layer; OPL, outer plexiform layer; ONL, outer nuclear layer; RPE, retinal pigment epithelium.

Organic cation transporter novel-type 1 is responsible for Ergo uptake and accumulation in eye tissues, and therefore we initially analyzed eye cross-sections to localize OCTN1 transporters (Figure 1 and Table 1). Total OCTN1 immunoreactivity in the whole eye was significantly different across the three animal groups (*p* = 0.002, Kruskal–Wallis H test) (Table 1). It was significantly high in WT controls compared with non-treated or Ergo-treated 5XFAD (*p* = 0.002 and *p* = 0.002, respectively Bonferroni corrected Dunn’s *post-hoc* test) (Figure 1T). Between anterior [mean ± standard error of mean (SEM), 1,835.1 ± 386.0] and posterior (1,238.3 ± 265.7) segments (as indicated in Figure 1S), total OCTN1 immunoreactivity was not significantly different (*p* = 0.19, Student’s *t*-test). OCTN1 immunoreactivity in anterior segments of the eye such as cornea, lens, and ciliary body, and in posterior segments of the eye such as retina and RPE/Choroid were separately compared across the three animal groups and summarized (Table 1 and Figures 1U, V). Total OCTN1 immunoreactivity in anterior segments were significantly different (*p* = 0.029, Kruskal–Wallis H test) across the three animal groups. OCTN1 expressions in anterior segments of the WT controls (Figures 1A, D, G), non-treated 5XFAD (Figures 1B, E, H) and Ergo-treated 5XFAD (Figures 1C, F, I) are illustrated. Between animal groups, it was significantly high in WT controls compared with non-treated 5XFAD (*p* = 0.015, Bonferroni corrected Dunn’s *post-hoc* test) (Figure 1U). Similarly, total OCTN1 immunoreactivity in posterior segments were significantly different (*p* = 0.029, Kruskal–Wallis H test) across the three animal groups. OCTN1 expressions in posterior segments of the WT controls (Figures 1J, M, P), non-treated 5XFAD (Figures 1K, N, Q), and Ergo-treated 5XFAD (Figures 1L, O, R) are illustrated. Between animal groups, it was significantly high in WT controls compared with Ergo-treated 5XFAD (*p* = 0.016, Bonferroni corrected Dunn’s *post-hoc* test) (Figure 1V). However, individual region-based analysis did not show any significant difference across the three animal groups (Table 1). Intergroup comparisons showed no significant difference in the level of expression of OCTN1 transporters in both anterior and posterior segments of the eye of Ergo-treated and non-treated 5XFAD mice.

**TABLE 1:** OCTN1 expression in the eye cross-sections.

Mouse models compared	Retinal cross-sections	Kruskal–Wallis H test (*P*)	Test statistics	Std. test statistics	Dunn’s *post-hoc* test	
					***P*^a^-value**	**Bonferroni corrected *P*-value**
5XFAD no-trt vs. WT con	Whole eye	0.002**	28.223	3.07	0.002**	0.006
5XFAD no-trt vs. 5XFAD trt			–0.33	–0.036	0.971	1.000
5XFAD trt vs. WT con			27.893	3.03	0.002**	0.007
5XFAD no-trt vs. WT con	Anterior segments	0.029*	14.646	2.42	0.015*	0.046
5XFAD no-trt vs. 5XFAD trt			–1.604	–0.27	0.791	1.000
5XFAD trt vs. WT con			13.042	2.16	0.031	0.093
5XFAD no-trt vs. WT con	Posterior segments	0.029*	15.188	2.18	0.029	0.088
5XFAD no-trt vs. 5XFAD trt			1.594	0.23	0.819	1.000
5XFAD trt vs. WT con			16.781	2.41	0.016*	0.048
5XFAD no-trt vs. WT con	Cornea	0.150	n/a		n/a	
5XFAD no-trt vs. 5XFAD trt						
5XFAD trt vs. WT con						
5XFAD no-trt vs. WT con	Ciliary body	0.36	n/a		n/a	
5XFAD no-trt vs. 5XFAD trt						
5XFAD trt vs. WT con						
5XFAD no-trt vs. WT con	Lens	0.199	n/a		n/a	
5XFAD no-trt vs. 5XFAD trt						
5XFAD trt vs. WT con						
5XFAD no-trt vs. WT con	Retina	0.117	n/a		n/a	
5XFAD no-trt vs. 5XFAD trt						
5XFAD trt vs. WT con						
5XFAD no-trt vs. WT con	RPE/Choroid	0.029*	8.0	2.26	0.024	0.071
5XFAD no-trt vs. 5XFAD trt			0.312	0.09	0.93	1.000
5XFAD trt vs. WT con			8.312	2.35	0.019	0.056

Pixel counts obtained for OCTN1 labeling in the eye cross-sections of age-matched WT (C57BL/6J) controls (*n* = 4), non-treated 5XFAD (*n* = 4) and 5XFAD Ergo-treated (*n* = 4) were compared using non-parametric one-way ANOVA test (Kruskal–Wallis H test, 2-sided) followed by Bonferroni corrected Dunn’s *post-hoc* test for multiple comparisons at a significance level of *p* < 0.05 indicated as *p < 0.05 and **p < 0.01. Orthogonal projections of z-stacks captured at 400x magnification were used for pixel count analysis. OCTN1, Organic novel cation transporter 1; WT, wildtype; con, controls; no-trt, Non-treated; trt, Ergo-treated; Std., standard deviation; *P^a^*, actual *P* value; RPE, retinal pigment epithelium.

### 3.2. Measurement of 6E10 immunoreactivity in the wholemount neuroretinas along with GFAP or IBA1 markers

**FIGURE 2:**
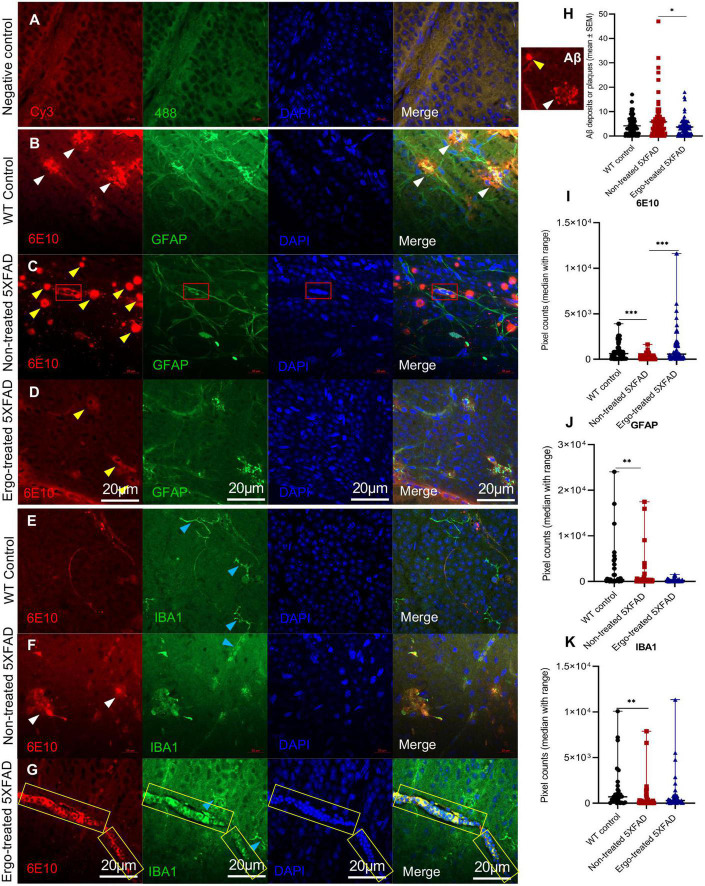
Colocalization of Aβ protein along with astrocytes or microglia/macrophage markers in the wholemount neuroretinas. **(A)** Negative control omitted for primary antibodies; double immunolabeling of **(B–D)** 6E10 and GFAP markers, and **(E–G)** 6E10 and IBA1 markers across the three animal groups are illustrated. 6E10(+)ve neuritic type Aβ plaques (white arrowheads) or large deposits (yellow arrowheads), GFAP+(ve) phagocytic astrocytes/PLS (red box), IBA1+(ve) microglia (blue arrowheads) or blood-derived macrophages (yellow box) are indicted. **(H)** Aβ plaque counts were compared across the three animal groups and illustrated using scatter plots. **(I–K)** 6E10, GFAP, and IBA1 immunoreactivities, respectively were compared across the three animal groups and illustrated using scatter plots. Significance levels of *p* < 0.05 are indicated as *p < 0.05, ^**^p < 0.01, and ^***^p < 0.001. Scale bar, 20 μm; bright red, 6E10; bright green, IBA1; blue, DAPI. WT, C57BL/6J wildtype; SEM, standard error of mean.

Colocalization of 6E10 and GFAP or IBA1 antibodies in the wholemounts are illustrated along with negative control (Figures 2A–G), and their immunoreactivity was compared across the three animal groups (Figures 2I–K and Table 2). Existence of neuritic type Aβ plaques surrounded by reactive astrocytes (Figure 2B, white arrowheads), large Aβ deposits (Figure 2C, yellow arrowheads), GFAP+(ve) phagocytic astrocytes/PLS (Figure 2C, red boxes), IBA1+(ve) microglia (Figures 2E–G, blue arrowheads) and phagocytic IBA1(+)ve blood-derived macrophages/PLS (Figure 2G, yellow boxes) in the wholemounts are indicated (details were given in the following paragraphs). Antibody labeling was predominantly detected on ILM/NFL and GCL, and thus orthogonal projections represented the immunoreactivity that were obtained from ILM/NFL and GCL. 6E10 immunoreactivity was significantly different across the three animal groups (*p* < 0.001, Kruskal–Wallis H test) (Table 2). Between groups, it was significantly high in Ergo-treated 5XFAD and WT controls (*p* < 0.001 and *p* < 0.001 respectively, Bonferroni corrected Dunn’s *post-hoc* test) compared with non-treated 5XFAD (Figure 2I). With respect to glial cell markers, GFAP and IBA1 immunoreactivities were significantly different across the three animal groups (*p* = 0.020 and *p* = 0.002, respectively, Kruskal–Wallis H test) (Table 2). However, between groups, GFAP and IBA1 immunoreactivities were significantly high in WT controls compared with non-treated 5XFAD (*p* = 0.006 and *p* = 0.001, respectively, Bonferroni corrected Dunn’s *post-hoc* test) (Figures 2J, K). Although it was statistically non-significant, GFAP immunoreactivity was reduced in the wholemounts Ergo-treated 5XFAD compared with WT controls (Figure 2J).

**TABLE 2:** Expression of 6E10, IBA1, and GFAP protein markers in the wholemount neuroretinas.

Mouse models compared	Wholemount neuroretinas	Kruskal–Wallis H test (*P*)	Test statistics	Std. test statistics	Dunn’s *post-hoc* test	
					***P*^a^-value**	**Bonferroni corrected *P*-value**
5XFAD no-trt vs. WT con	6E10	<0.001***	43.869	3.93	<0.001***	<0.001
5XFAD no-trt vs. 5XFAD trt			–59.264	–5.95	<0.001***	<0.001
5XFAD trt vs. WT con			–15.395	–1.41	0.159	0.477
5XFAD no-trt vs. WT con	IBA1	0.002**	27.214	3.44	0.001**	0.002
5XFAD no-trt vs. 5XFAD trt			–15.535	–2.2	0.028	0.083
5XFAD trt vs. WT con			11.679	1.51	0.132	0.395
5XFAD no-trt vs. WT con	GFAP	0.020*	21.566	2.75	0.006**	0.018
5XFAD no-trt vs. 5XFAD trt			–12.647	–1.72	0.086	0.259
5XFAD trt vs. WT con			8.919	1.11	0.268	0.803

Pixel counts obtained for 6E10, IBA1, and GFAP labeling in the wholemount neuroretinas of age-matched WT (C57BL/6J) controls (*n* = 8), non-treated 5XFAD (*n* = 9), and Ergo-treated 5XFAD (*n* = 9) were compared using Kruskal–Wallis H test (2-sided) followed by Bonferroni corrected Dunn’s *post-hoc* test for multiple comparisons at a significance level of *p* < 0.05 indicated as *p < 0.05, ^**^p < 0.01, and ^***^p < 0.001. Orthogonal projections of z-stacks captured at 200x magnification were used for pixel count analysis. WT, Wildtype; con, controls; trt, Ergo-treated; no-trt, Non-treated; Std., standard deviation; *P^a^*, actual *P* value.

**FIGURE 3:**
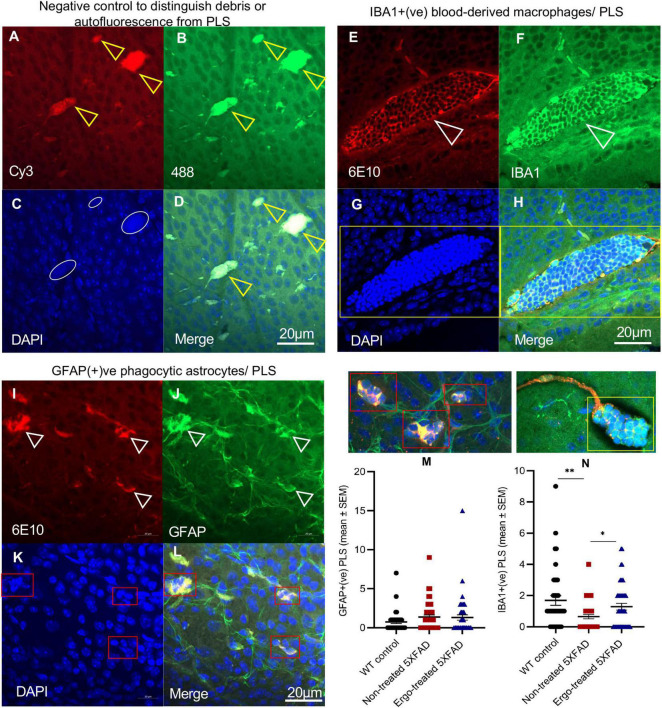
Evidence for Aβ clearance by phagosome like structures on the surface of wholemount neuroretinas. **(A–D)** Negative control omitted for primary antibodies to distinguish debris or autofluorescence bodies (yellow arrowheads), **(E–H)** IBA1+(ve) phagocytic blood-derived macrophages or PLS (white arrowheads), and **(I–L)** GFAP+(ve) phagocytic astrocytes or PLS (white arrowheads) are illustrated on the surface of wholemount neuroretinas. **(C)** White circles in negative control show poor uptake of DAPI which distinguish the PLS from debris or autofluorescence based on DAPI(+)ve clumps of nuclei (**G,K**, indicated by yellow and red boxes). Mean counts of GFAP(+)ve **(M)** and IBA1(+)ve **(N)** PLSs as indicated by red and yellow boxes, respectively were compared across the three animal groups and illustrated using scatter plots. Significance levels of *p* < 0.05 are indicated as *p < 0.05 and ^**^p < 0.01. Scale bar, 20 μm; bright red, 6E10; bright green, IBA1/GFAP; blue, DAPI. SEM, standard error of mean; PLS, phagosome like structure.

**FIGURE 4:**
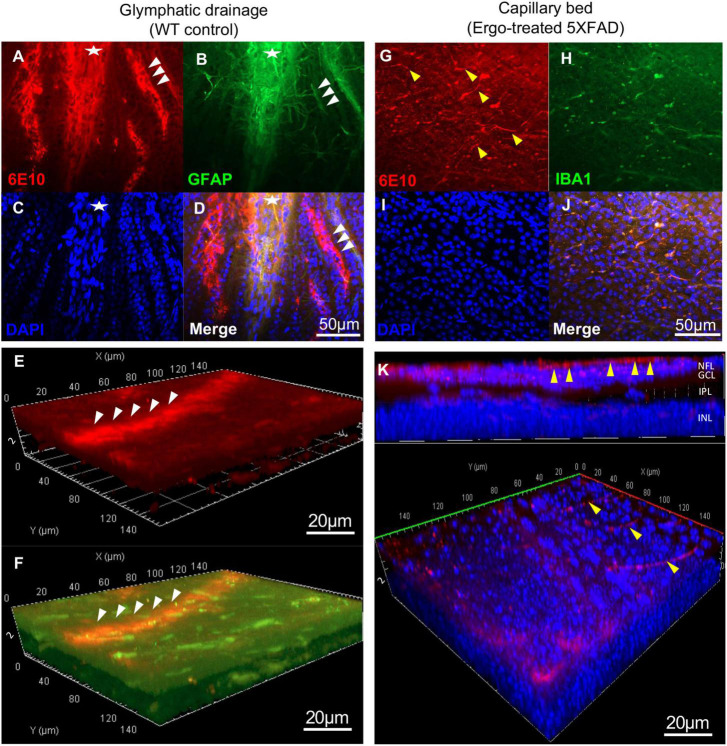
Evidence for Aβ clearance through glymphatic and perivascular drainage systems on the surface of wholemount neuroretinas. 6E10 and GFAP colocalization revealed the existence of glymphatic drainages on the surface of wholemount neuroretinas. **(A–D)** Glymphatic drainage mediated by tightly attached astrocytic end-feet (white arrowheads) toward optic nerve (white asterisk), and the **(E,F)** 3D projections demonstrated its existence at surface level. **(G–J)** Aβ influx into the perivascular drainage by terminal end of the capillary bed (yellow arrowheads) on the surface of wholemount neuroretinas. **(K)** 3D projections demonstrated the existence of capillary bed involved in Aβ clearance at surface level. Scale bars, 50 μm and 20 μm; bright red, 6E10; bright green, GFAP; blue, DAPI. NFL, nerve fiber layer; GCL, ganglion cell layer; IPL, inner plexiform layer; INL, inner nuclear layer.

A significantly high level of 6E10 immunoreactivity on the surface of wholemounts of Ergo-treated 5XFAD and WT controls suggests the existence of effective Aβ clearance mechanisms. This could be possibly contributed by various systems and cells that involved in Aβ clearance such as phagocytic IBA1+(ve) resident and/or blood-derived macrophages, GFAP+(ve) phagocytic astrocytes/PLS, and glymphatic and perivascular drainages. To support the above suggestion, we provided the evidence for the existence of effective Aβ clearance mechanisms on the surface of wholemounts (Figures 3, 4). Negative control slide was prepared without primary antibodies to distinguish the debris or autofluorescence bodies (Figures 3A–D) in which DAPI uptake was poor (white circles in Figure 3D). IBA1(+)ve PLS (Figures 3E–H, yellow box and Supplementary Videos 1, 2) and GFAP(+)ve PLS (Figures 3I–L, red boxes and Supplementary Videos 3, 4) are found on top of the GCL. Based on triple positive staining, functional PLS (Figures 3E–L) were distinguished from debris or autofluorescence bodies (Figures 3A–D). However, the size of the functional PLS [GFAP+(ve) or IBA1+(ve)] was varied across the three animal groups (Supplementary Image 1).

Besides, to confirm the existence of effective Aβ clearance stimulated by Ergo treatment, a semi-quantification protocol was adapted to count 6E10+(ve) Aβ plaques or large deposits, GFAP+(ve) PLS and IBA1+(ve) PLS on the surface of wholemount neuroretinas manually (Figures 2H, 3M, N). Mean counts (mean ± SEM) were significantly low in Ergo-treated (3.74 ± 0.37) compared with non-treated (5.83 ± 0.75) 5XFAD (*p* = 0.036, Games-Howell *post-hoc* test) (Figure 2H). Whereas mean counts of 6E10+(ve) Aβ deposits in WT controls (4.17 ± 0.43) were non-significant when compared with Ergo-treated or non-treated 5XFAD. Mean counts of GFAP+(ve) PLS were 1.32 ± 0.34, 1.37 ± 0.27, and 0.76 ± 0.22 in Ergo-treated 5XFAD, non-treated 5XFAD and WT controls, respectively though it was statistically non-significant across the three animal groups (*p* = 0.308, one-way ANOVA test) [Figure 3M, each red box was counted as one GFAP+(ve) PLS]. Mean counts of IBA1+(ve) PLS were significantly high in Ergo-treated 5XFAD (1.29 ± 0.21) and WT controls (1.69 ± 0.31) compared with non-treated 5XFAD (0.65 ± 0.14) (*p* = 0.038, and *p* = 0.009, respectively, Games-Howell *post-hoc* test) [Figure 3N, each yellow box was counted as one IBA1+(ve) PLS]. GFAP+(ve) or IBA1+(ve) PLSs were identified by 6E10+(ve) Aβ deposits that were engulfed or surrounded by DAPI+(ve) clumps of nuclei.

In addition to PLS, existence of glymphatic drainage, mediated by tightly attached astrocytic end-feet (white arrowheads) toward proximal end of the optic nerve (white asterisk) was identified on the surface of wholemounts (Figures 4A–D and Supplementary Videos 5, 6). Its existence at the surface level was demonstrated *via* 3D projections (Figures 4E, F, white arrowheads). Similarly, terminal end of the capillary beds (yellow arrowheads), facilitating Aβ influx into perivascular drainage were identified on the surface of wholemounts as demonstrated *via* 3D projections (Figures 4G–K and Supplementary Video 7).Visually, Aβ clearance *via* glymphatic drainage mediated by astrocytes and perivascular drainage mediated by capillary beds were mostly identified in WT controls and Ergo-treated 5XFAD, respectively. However, their existence was also noticed in the remaining animal groups in varying degrees (Supplementary Image 2).

### 3.3. Measurement of 6E10 immunoreactivity in the retinal cross-sections along with AQP4 or GFAP or IBA1 markers

**FIGURE 5:**
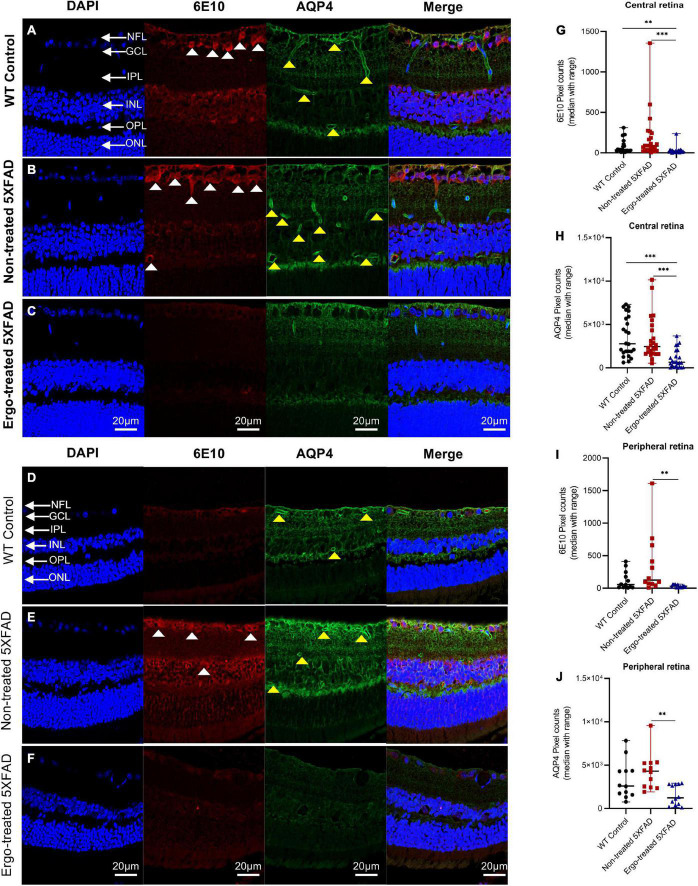
Colocalization of 6E10 and AQP4 protein markers in the retinal cross-sections. Expression pattern of 6E10 and AQP4 markers in the central and peripheral regions of **(A,D)** WT (C57BL/6J) controls, **(B,E)** non-treated 5XFAD, and **(C,F)** Ergo-treated 5XFAD are illustrated. White arrowheads indicate intraneuronal accumulation of 6E10(+)ve Aβ proteins, and the yellow arrowheads indicate AQP4(+)ve water channels across the retinal layers. 6E10 and AQP4 immunoreactivities in the **(G,H)** central, and **(I,J)** peripheral retinas were compared across the three animal groups and illustrated using scatter plots. Significance levels of *p* < 0.05 are indicated as ^**^p < 0.01 and ^***^p < 0.001. Scale bar, 20 μm; bright red, 6E10; bright green, AQP4. NFL, nerve fiber layer; GCL, ganglion cell layer; IPL, inner plexiform layer; INL, inner nuclear layer; OPL, outer plexiform layer; ONL, outer nuclear layer.

In addition to wholemounts staining, eye cross-sections were used to screen the 6E10, AQP4, GFAP, and IBA1 expression patterns across the retinal layers (Tables 3, 4). Colocalization of 6E10 and AQP4 markers in the central and far peripheral retinal cross-sections are illustrated (Figures 5A–F). 6E10 and AQP4 immunoreactivities were significantly different across the three animal groups (*p* < 0.001 and *p* < 0.001, respectively, Kruskal–Wallis H test) (Table 3). Between groups, 6E10 immunoreactivity was significantly low in Ergo-treated 5XFAD compared with WT controls or non-treated 5XFAD (*p* = 0.005 and *p* < 0.001, respectively, Bonferroni corrected Dunn’s *post-hoc* test) (Table 3). Similarly, between groups, AQP4 immunoreactivity was significantly low in Ergo-treated 5XFAD compared with WT controls or non-treated 5XFAD (*p* < 0.001 and *p* < 0.001, respectively, Bonferroni corrected Dunn’s *post-hoc* test) (Table 3). 6E10 and AQP4 expressions were further analyzed in detail for central and peripheral retinas (Table 3 and Figures 5G, H). In both central and peripheral retinas, immunoreactivities were significantly low for 6E10 (*p* < 0.001 and *p* = 0.003, respectively, Bonferroni corrected Dunn’s *post-hoc* test) and AQP4 (*p* < 0.001 and *p* = 0.002, respectively, Bonferroni corrected Dunn’s *post-hoc* test) in Ergo-treated compared with non-treated 5XFAD. Whereas 6E10 and AQP4 immunoreactivities were significantly low only in the central retina of Ergo-treated 5XFAD compared with WT controls (*p* = 0.004 and *p* < 0.001, respectively, Bonferroni corrected Dunn’s *post-hoc* test) (Table 3).

**TABLE 3:** Expression of 6E10 and AQP4 protein markers in the retinal cross-sections.

Mouse models compared	Retinal cross-sections	Kruskal–Wallis H test (*P*)	Test statistics	Std. test statistics	Dunn’s *post-hoc* test	
					***P*^a^-value**	**Bonferroni corrected *P*-value**
5XFAD no-trt vs. WT con	6E10 (whole)	<0.001***	–16.799	–2.28	0.022	0.067
5XFAD no-trt vs. 5XFAD trt			37.625	5.193	<0.001***	<0.001
5XFAD trt vs. WT con			20.826	2.83	0.005**	0.014
5XFAD no-trt vs. WT con	6E10 (central)	<0.001***	–8.398	–1.4	0.162	0.486
5XFAD no-trt vs. 5XFAD trt			25.479	4.34	<0.001***	<0.001
5XFAD trt vs. WT con			17.081	2.84	0.004**	0.013
5XFAD no-trt vs. WT con	6E10 (peripheral)	0.011*	–8.208	-1.91	0.056	0.169
5XFAD no-trt vs. 5XFAD trt			12.792	2.97	0.003**	0.009
5XFAD trt vs. WT con			4.583	1.07	0.287	0.860
5XFAD no-trt vs. WT con	AQP4 (whole)	<0.001***	–2.917	-0.39	0.693	1.000
5XFAD no-trt vs. 5XFAD trt			37.417	5.07	<0.001***	<0.001
5XFAD trt vs. WT con			34.5	4.67	<0.001***	<0.001
5XFAD no-trt vs. WT con	AQP4 (central)	<0.001***	1.833	0.3	0.762	1.000
5XFAD no-trt vs. 5XFAD trt			24.583	4.07	<0.001***	<0.001
5XFAD trt vs. WT con			26.417	4.373	<0.001***	<0.001
5XFAD no-trt vs. WT con	AQP4 (peripheral)	0.007**	–5.5	–1.28	0.201	0.603
5XFAD no-trt vs. 5XFAD trt			13.5	3.14	0.002**	0.005
5XFAD trt vs. WT con			8.0	1.86	0.063	0.189

Pixel counts obtained for 6E10 and AQP4 labeling in the retinal cross-sections of age-matched WT (C57BL/6J) controls (*n* = 6), non-treated 5XFAD (*n* = 6) and Ergo-treated 5XFAD (*n* = 6) were compared using Kruskal–Wallis H test (2-sided) followed by Bonferroni corrected Dunn’s post hoc test for multiple comparisons at a significance level of *p* < 0.05 indicated as *p < 0.05, ^**^p < 0.01, and ^***^p < 0.001. Images captured at 200x magnification were used for pixel count analysis. WT, Wildtype; con, controls; trt, Ergo-treated; no-trt, Non-treated; Std., standard deviation; *P^a^*, actual *P* value.

**TABLE 4:** Expression of IBA1 and GFAP protein markers in the retinal cross-sections.

Mouse models compared	Retinal cross-sections	Kruskal–Wallis H test (*P*)	Test statistics	Std. test statistics	Dunn’s *post-hoc* test	
					***P*^a^-value**	**Bonferroni corrected *P*-value**
5XFAD no-trt vs. WT con	IBA1 (whole)	0.873	n/a		n/a	
5XFAD no-trt vs. 5XFAD trt						
5XFAD trt vs. WT con						
5XFAD no-trt vs. WT con	IBA1 (central)	0.93	n/a		n/a	
5XFAD no-trt vs. 5XFAD trt						
5XFAD trt vs. WT con						
5XFAD no-trt vs. WT con	IBA1 (peripheral)	0.928	n/a		n/a	
5XFAD no-trt vs. 5XFAD trt						
5XFAD trt vs. WT con						
5XFAD no-trt vs. WT con	GFAP (whole)	<0.001***	17.646	2.92	0.003**	0.010
5XFAD no-trt vs. 5XFAD trt			19.708	3.26	0.001**	0.003
5XFAD trt vs. WT con			37.354	6.18	<0.001***	<0.001
5XFAD no-trt vs. WT con	GFAP (central)	0.001**	8.542	1.97	0.047	0.141
5XFAD no-trt vs. 5XFAD trt			8.167	1.9	0.058	0.173
5XFAD trt vs. WT con			16.708	3.88	<0.001***	<0.001
5XFAD no-trt vs. WT con	GFAP (peripheral)	<0.001***	8.917	2.07	0.038	0.114
5XFAD no-trt vs. 5XFAD trt			11.667	2.712	0.007**	0.020
5XFAD trt vs. WT con			20.583	4.79	<0.001***	<0.001

Pixel counts obtained for 6E10 and AQP4 labeling in the retinal cross-sections of age-matched WT (C57BL/6J) controls (*n* = 6), non-treated 5XFAD (*n* = 6) and Ergo-treated 5XFAD (*n* = 6) were compared using Kruskal–Wallis H test (2-sided) followed by Bonferroni corrected Dunn’s post hoc test for multiple comparisons at a significance level of *p* < 0.05 indicated as **p < 0.01 and ***p < 0.001. Orthogonal projections of z-stacks captured at 200x magnification were used for pixel count analysis. WT, Wildtype; con, controls; trt, Ergo-treated; no-trt, Non-treated; Std., standard deviation; *P^a^*, actual *P* value.

**FIGURE 6:**
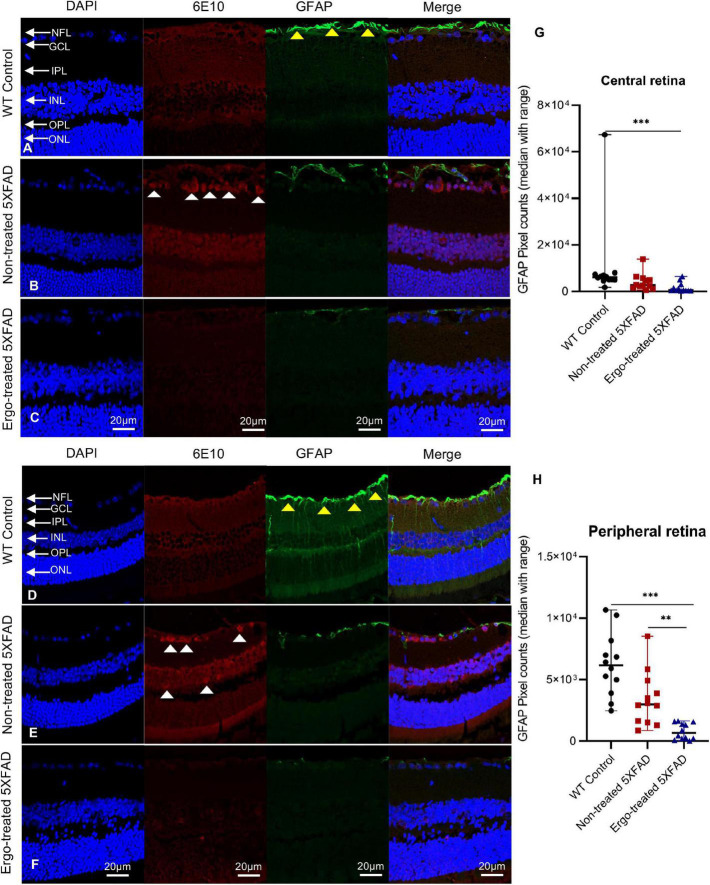
Colocalization of 6E10 and GFAP protein markers in the retinal cross-sections. Expression pattern of 6E10 and GFAP markers in the central and peripheral regions of **(A,D)** WT (C57BL/6J) controls, **(B,E)** non-treated 5XFAD, and **(C,F)** Ergo-treated 5XFAD are illustrated. White arrowheads indicate intraneuronal accumulation of 6E10(+)ve Aβ proteins, and yellow arrowheads indicate GFAP(+)ve astrocytes, predominantly localized in NFL. GFAP immunoreactivity in the **(G)** central, and **(H)** peripheral retinas were compared across the three animal groups and illustrated using scatter plots. Significance levels of *p* < 0.05 are indicated as ^**^p < 0.01 and ^***^p < 0.001. Scale bar, 20 μm; bright red, 6E10; bright green, GFAP. NFL, nerve fiber layer; GCL, ganglion cell layer; IPL, inner plexiform layer; INL, inner nuclear layer; OPL, outer plexiform layer; ONL, outer nuclear layer.

**FIGURE 7:**
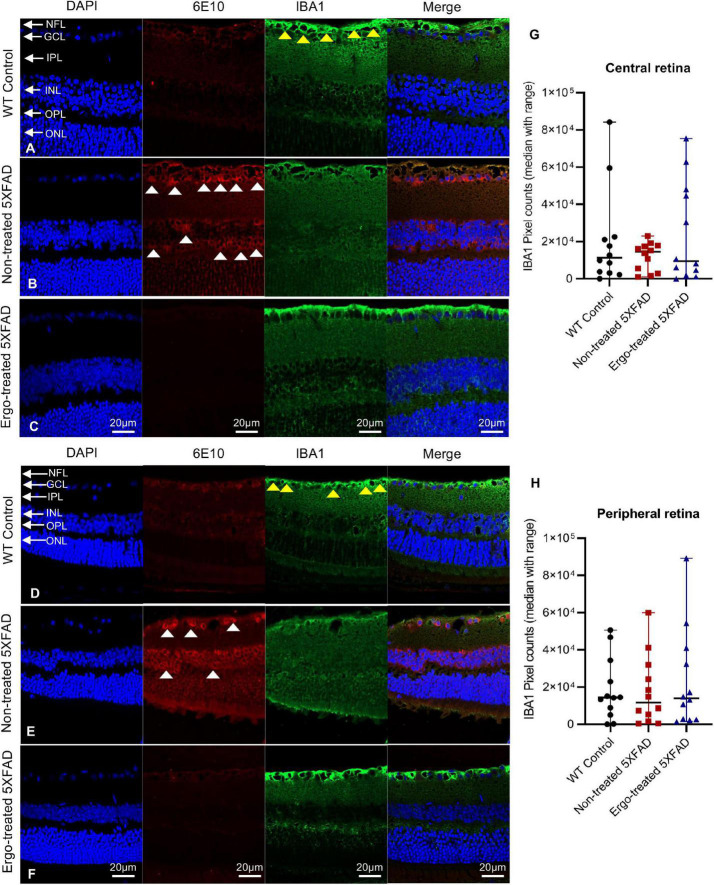
Colocalization of 6E10 and IBA1 protein markers in the retinal cross-sections. Expression pattern of 6E10 and IBA1 markers in the central and peripheral regions of **(A,D)** WT (C57BL/6J) controls, **(B,E)** non-treated 5XFAD, and **(C,F)** Ergo-treated 5XFAD are illustrated. White arrowheads indicate intraneuronal accumulation of 6E10(+)ve Aβ proteins, and yellow arrowheads indicate IBA1(+)ve microglia or macrophages, predominantly localized in NFL. IBA1 immunoreactivity in the **(G)** central and **(H)** peripheral retinas were compared across the three animal groups and illustrated using scatter plots. IBA1 immunoreactivity was non-significant across the three animal groups in central and peripheral retinas. Scale bar, 20 μm; bright red, 6E10; bright green, IBA1. NFL, nerve fiber layer; GCL, ganglion cell layer; IPL, inner plexiform layer; INL, inner nuclear layer; OPL, outer plexiform layer; ONL, outer nuclear layer.

GFAP immunoreactivity in the retinal cross-sections were significantly different across the three animal groups (*p* < 0.001, Kruskal–Wallis H test) whereas IBA1 immunoreactivity was not significantly different (*p* = 0.873, Kruskal–Wallis H test) (Table 4). Colocalization of 6E10 and GFAP or IBA1 markers in the central and far peripheral retinal cross-sections are illustrated (Figures 6A–F, 7A–F, respectively). GFAP immunoreactivity was significantly low in Ergo-treated or non-treated 5XFAD compared with WT controls (*p* < 0.001 and *p* = 0.003, respectively, Bonferroni corrected Dunn’s *post-hoc* test). Moreover, between Ergo-treated and non-treated 5XFAD, GFAP immunoreactivity was significantly high in non-treated 5XFAD (*p* = 0.001, Bonferroni corrected Dunn’s *post-hoc* test). GFAP expression was further analyzed in detail for central and peripheral retinas (Table 4 and Figures 6G, H). In both central and peripheral retinas, immunoreactivities were significantly low in Ergo-treated 5XFAD compared with WT controls (*p* < 0.001 and *p* < 0.001, respectively Bonferroni corrected Dunn’s *post-hoc* test). Whereas, between Ergo-treated and non-treated 5XFAD, GFAP immunoreactivity was significantly low only in the peripheral retina of Ergo-treated 5XFAD (*p* = 0.007, Bonferroni corrected Dunn’s *post-hoc* test). Further, detail analysis of IBA1 immunoreactivity in central and peripheral retinas did not show any significant difference across the three animal groups (Figures 7G, H).

**FIGURE 8:**
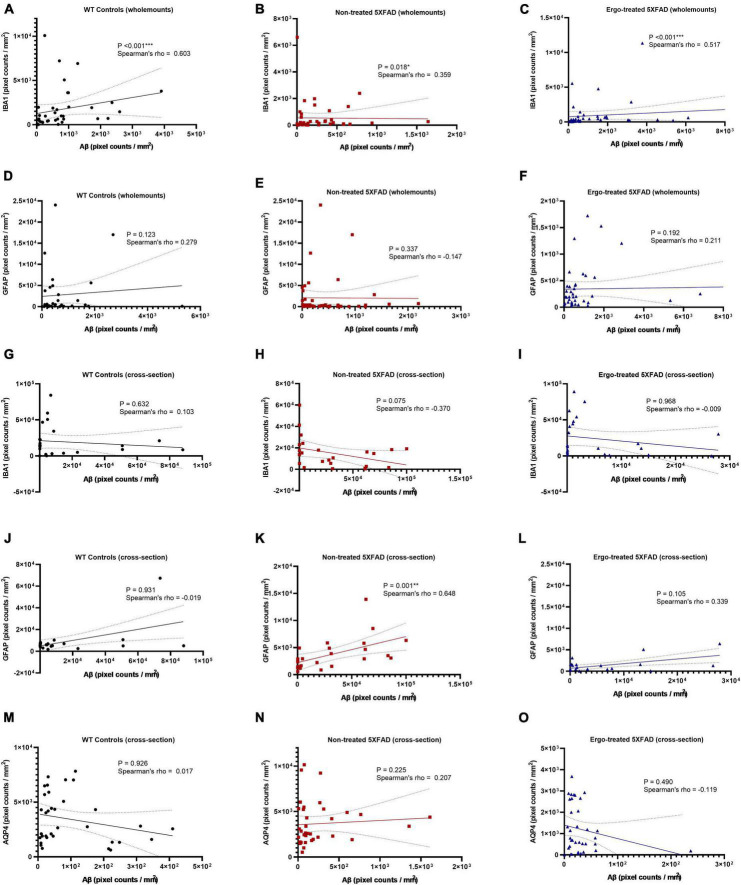
Correlation between 6E10 and IBA1 or GFAP or AQP4 protein markers in the wholemount neuroretinas and retinal cross-sections. Spearman’s rank-order correlation test was conducted to see the association between protein markers based on the plane of view (surface vs. cross-sections). The strength of association between 6E10 and IBA1 expressions in the hemi-retinal wholemounts **(A–C)**, and retinal cross-sections **(G–I)**; between 6E10 and GFAP expressions in the hemi-retinal wholemounts **(D–F)**, and retinal cross-sections **(J–L)**; and between 6E10 and AQP4 expressions in the retinal cross-sections **(M–O)** are illustrated. Significance levels of *p* < 0.05 are indicated as *p < 0.05, **p < 0.01, and ***p < 0.001.

In addition to multiple comparisons, Spearman’s rank-order correlation test was conducted to see the strength of association between 6E10 and IBA1 or 6E10 and GFAP expressions in the hemi-retinal wholemounts (Figures 8A–F). Correlation coefficients (ρ) obtained between 6E10 and IBA1 expressions were significant and positively related in all three groups, however the associations were stronger in WT controls (*p* < 0.001, ρ = 0.603) followed by Ergo-treated 5XFAD (*p* < 0.001, ρ = 0.517) compared with non-treated 5XFAD (*p* = 0.018, ρ = 0.359) (Figures 8A–C). Whereas ρ values obtained between 6E10 and GFAP expressions were statistically non-significant in all three animal groups (Figures 8D–F). Spearman’s correlation test was also conducted to see the strength of association between 6E10 and IBA1 or GFAP or AQP4 expressions in the retinal cross-sections (Figures 8G–O). Between 6E10 and IBA1 or 6E10 and AQP4 expressions, ρ values were statistically non-significant in all three animal groups (Figures 8G–I, M–O). Similarly, between 6E10 and GFAP expressions, ρ values were statistically non-significant in WT controls and Ergo-treated 5XFAD (Figures 8J, L), whereas it was significant and positively correlated in non-treated 5XFAD (*p* = 0.001, ρ = 0.648) (Figure 8K).

## 4. Discussion

Despite enormous scientific attempts, the treatment of AD has remained symptom-based and no single effective therapy for AD has been approved. Nutritional factors could have an impact in preventing chronic diseases, ensuring healthy aging (Wijesinghe et al., 2016), however, the usefulness of such a resource relies on documented evidence of the effects. Neuroprotective capabilities of Ergo have been demonstrated in a range of *in vitro* and *in vivo* studies, however underlying mechanism remains unclear. Recently, we reported that longitudinal consumption of Ergo reduced Aβ plaques in the brain cross-sections, oxidative stress, and rescued glucose metabolism in a 5XFAD mouse model (Whitmore et al., 2022). In our previous work, an efficient and reproducible preparation of [^11^C]-labeled Ergo PET radioligand to detect threshold levels of oxidative stress in a preclinical animal model of AD was also reported (Behof et al., 2022).

Since the Ergo uptake is dependent on OCTN1 transporters, we examined eye cross-sections of WT controls, non-treated and Ergo-treated 5XFAD to localize the OCTN1 transporters. OCTN1 expression significantly reduced in the retinas of 5XFAD compared with age-matched WT controls (Figures 1T–V). To our knowledge, this is the first study that localized OCTN1 transporters in different anatomical structures of the eye. Notably, OCTN1 expression was weak in the RPE/Choroid regions across the strains studied, in contrast, the ciliary body, which is an extension of the anterior choroid, elicited strong immunofluorescence to OCTN1 labeling, and a higher level of expression of OCTN1 transporters in the anterior segments of the eye implying the importance of Ergo uptake against oxygen tension due to ultraviolet (UV) light exposures (Shires et al., 1997). In our study, total OCTN1 immunoreactivity was high in the anterior compared with posterior segments, however it was not significantly different (*p* = 0.19). It is surprising that the outer retina (photoreceptors and RPE), also susceptible to high oxidative stress, did not demonstrate stronger immunoreactivity to OCTN1 transporters across the strains (Datta et al., 2017). In sum, our study demonstrated the significant exhaustion of OCTN1 transporters in the eye which facilitate the Ergo uptake and its subsequent antioxidant and/or anti-inflammatory effects against Aβ build up in a 5XFAD mouse model.

Aβ accumulation has been hypothesized to result from an imbalance between Aβ production and clearance; indeed, Aβ clearance seems to be impaired in both early and late forms of AD (Tarasoff-Conway et al., 2015). Aβ is removed from the brain by various overlapping and interacting clearance systems. Moreover, dysfunction of innate immunity, mainly involving microglia and peripheral myeloid cells, has been suggested as a critical reason in the development of AD (Bradshaw et al., 2013; Vasquez et al., 2013; Carmona et al., 2018). In our work, 6E10+(ve) signals obtained from the surface of hemi-retinal wholemounts were significantly high in Ergo-treated 5XFAD, followed by WT controls (Table 2) suggesting that Aβ efflux might contribute to its clearance and therefore 6E10 immunoreactivity is significantly increased on the surface. Since the insoluble Aβ deposit is the major pathological hallmark in AD pathogenesis, we manually counted the Aβ plaques or large deposits across the three animal groups. Mean counts were significantly low in Ergo-treated compared with non-treated 5XFAD (Figure 2H), but it was non-significant between WT controls and Ergo-treated or non-treated 5XFAD. Moreover, 6E10 immunoreactivity in the eye cross-sections demonstrated a significantly higher level of Aβ accumulations within retinal layers in non-treated 5XFAD, followed by WT controls when compared with Ergo-treated 5XFAD (Table 3 and Figures 5G, I). Based on the above results, we conclude that Aβ clearance process was effective in Ergo-treated 5XFAD, followed by WT controls. This is possibly contributed by involvement of various systems and cells such as IBA1+(ve) phagocytic resident or blood-derived macrophages (Figures 2G, 3E–H, Supplementary Images 1E, G, and Supplementary Videos 1, 2), GFAP+(ve) phagocytic astrocytes or PLS (Figures 2C, 3I–L, Supplementary Images 1B–D, and Supplementary Videos 3, 4), glymphatic drainage (Figures 4A–F, Supplementary Images 2B, C, and Supplementary Videos 5, 6), and the terminal end of the capillary bed (Figures 4G–K, Supplementary Images 2D, E, and Supplementary Video 7; Iliff et al., 2013; Zhao et al., 2015; Konishi et al., 2020).

Microglia are considered as the principal phagocytes in CNS (Wolf et al., 2017); they engulf dying or dead cells depending on the situation. Particularly, in the developing CNS, a number of unhealthy or misconnected neurons die and they are cleared by microglia (Oppenheim, 1991; Rigato et al., 2011). In addition, a large number of cellular debris generated upon neuronal injury is scavenged by reactive microglia (Sierra et al., 2013) and failure to clear such debris has detrimental effects on surrounding neural tissues. Impairment of microglial phagocytosis can occur in certain conditions, such as aging and injury (Abiega et al., 2016; Pluvinage et al., 2019). Reduced Aβ uptake capacity of microglia in the brain has been suggested as a major cause for AD as the microglial cells are capable of remodeling and enhancing the clearance of Aβ plaques (Kiyota et al., 2009; Chakrabarty et al., 2010). In our work, hemi-retinal wholemounts and the retinal cross-sections showed low level of IBA1 immunoreactivity in non-treated 5XFAD compared with WT controls or Ergo-treated 5XFAD (Figures 2K, 7G, H). The hemi-retinal wholemount analyses revealed a significant reduction in IBA1 immunoreactivity in non-treated 5XFAD compared with WT controls (Table 2). This may indicate that the functionality of microglia in non-treated 5XFAD is exhausted due to the relative “overload” of Aβ. However, it was comparable between WT controls and Ergo-treated 5XFAD, suggesting the possible contribution of IBA1+(ve) blood-derived macrophages for increased IBA1 immunoreactivity in Ergo-treated 5XFAD (Figures 3E–H and Supplementary Videos 1, 2). Zhao et al.’s (2015) study identified the involvement of retinal IBA1(+)ve microglia in inherited retinal degeneration where microglia infiltrate into the ONL and the multiple activated ameboid microglia form phagosomes and undertake phagocytosis of rods. Our semi-quantification study in wholemounts demonstrated a significantly high level of IBA1(+)ve PLS on top of the GCL of Ergo-treated 5XFAD and WT controls compared with non-treated 5XFAD (Figure 3N). This is further confirmed by correlation test in which wholemount data generated strong positive correlation between 6E10 and IBA1 expressions in WT controls (Figure 8A) and Ergo-treated 5XFAD (Figure 8C) compared with non-treated 5XFAD (Figure 8B), supporting the concept of effective Aβ clearance by resident microglia or blood-derived macrophages. Moreover, IBA1+(ve) PLSs are visually larger in size in Ergo-treated 5XFAD compared with WT controls (Supplementary Images 1E, G, respectively). This could possibly contribute to increased IBA1 immunoreactivity in wholemounts (Figure 2K) and ILM/NFL of the retinal cross-sections (Figures 7G, H) of Ergo-treated 5XFAD vs. WT controls, however, it was statistically non-significant.

In addition to microglia, phagocytic astrocytes involved in the elimination of debris and synaptic remodeling have been reported (Morizawa et al., 2017; Konishi et al., 2020). Konishi et al. (2020) suggest that astrocytes stand by in case of microglial impairment and this compensatory mechanism may be important for the maintenance and prolongation of healthy CNS. In transgenic AD mouse models, profound astrodegeneration occurs at the early stages of AD progression (Verkhratsky et al., 2019). At the early (i.e., pre-plaque) stages of the AD, astrocytes in the entorhinal cortex, prefrontal cortex, and hippocampus demonstrate signs of morphological atrophy (Verkhratsky et al., 2019). At the later stages of AD pathology, formation of plaques, and accumulation of extracellular Aβ initiates reactive astrogliosis. Numerous hypertrophic astrocytes accumulate exclusively around senile plaques and Aβ inundated blood vessels (Verkhratsky et al., 2019). In our study, GFAP expression was significantly different across the three animal groups in both wholemounts and cross-sections (*p* = 0.020 and *p* < 0.001, respectively, Kruskal–Wallis H test). GFAP immunoreactivity was significantly high in WT controls compared with non-treated or Ergo-treated 5XFAD. Between Ergo-treated and non-treated 5XFAD, GFAP immunoreactivity was non-significant in wholemounts (Figure 2J), whereas it was significantly high in the retinal cross-sections of non-treated 5XFAD (Table 4). Between central and peripheral retinas, 6E10 and GFAP immunoreactivities were significantly high in the peripheral retinas of non-treated 5XFAD compared with Ergo-treated 5XFAD (Figures 5I, 6H). Thus, it confirms the increased level of Aβ accumulations across the peripheral retinal layers in non-treated 5XFAD as reflected *via* strong GFAP immunoreactivity by reactive astrocytes. Retinal cross-section study also showed a significant positive correlation between 6E10 and GFAP expressions in non-treated 5XFAD (Figure 8K), supporting the literatures that colocalize Aβ plaques with reactive astrocytes in the human brain studies (Verkhratsky et al., 2016). Moreover, 6E10 and GFAP colocalization reveals the evidence for the presence of GFAP+(ve) phagocytic astrocytes/PLS (Figures 2C, 3I–L, Supplementary Images 1B–D, and Supplementary Videos 3, 4) on the surface of wholemount neuroretinas, although the number of GFAP(+)ve PLSs were statistically non-significant across the three animal groups (Figure 3M).

6E10 and GFAP colocalization also demonstrated the existence of glymphatic drainage mediated by astrocytic end-feet on the surface of wholemount neuroretinas (Figures 4A–F, Supplementary Images 2B, C, and Supplementary Videos 5, 6). The glymphatic system, which relies on astrocytes removes the brain’s “garbage” by moving large amounts of cerebrospinal fluid (CSF) quickly through the brain and sweeping the waste from interstitial spaces (Iliff et al., 2013; Xie et al., 2013). The failure of the glymphatic system in aging might contribute to the accumulation of misfolded and hyperphosphorylated proteins and thereby render the brain more vulnerable to developing a neurodegenerative pathology or perhaps escalate the progression of cognitive dysfunction (Jessen et al., 2015). Existence of ocular glymphatic waste clearance system *via* the proximal optic nerve has also been demonstrated in rodents (Wang et al., 2020). Astrocytes play a key role in which astrocytic end-feet and their dense expression of AQP4 water channel promotes fluid exchange between perivascular spaces and the neuropil (Mogensen et al., 2021). Wang et al. (2020) showed that Aβ was cleared from the retina and vitreous *via* a pathway dependent on glial water channel AQP4 and driven by the ocular-cranial pressure difference.

Since AQP4 water channels are an integral part of glymphatic system, we checked the AQP4 expression levels in the retinal cross-sections using double immunolabeling technique with 6E10 antibody. Pixel count analysis showed a significant difference in AQP4 expression across the three animal groups (Table 3). Most importantly, AQP4 immunoreactivity was significantly reduced in Ergo-treated 5XFAD mice compared with non-treated 5XFAD or WT controls. Significantly reduced 6E10 immunoreactivity in both central and peripheral retinas of Ergo-treated 5XFAD compared with non-treated 5XFAD confirmed low level of Aβ accumulations across the retinal layers which do not require AQP4 water channels for Aβ clearance. On the other hand, significantly increased 6E10 immunoreactivity in both central and peripheral retinas of non-treated 5XFAD compared with Ergo-treated 5XFAD confirmed high level of Aβ accumulations across the retinal layers which require AQP4 water channels for Aβ clearance. Although AQP4 immunoreactivity was significantly high in non-treated 5XFAD, high level of Aβ accumulations across the retinal layers suspect the existence of defective or depolarized AQP4 water channels in these samples. Between WT controls and Ergo-treated 5XFAD, 6E10 (Figure 5G), GFAP (Figure 6G), and AQP4 (Figure 5H) immunoreactivities were significantly high in the central retina of WT controls whereas it was comparable in peripheral retina (Figures 5I, J) except for GFAP (Figure 6H). Increased Aβ accumulations within central retina of WT controls could possibly be associated with the efficiency of drainage systems such as glymphatic vs. perivascular in WT controls vs. Ergo-treated 5XFAD, respectively (Figure 4 and Supplementary Image 2). Further, AQP4 mislocalization has been reported as a phenotype in AD to contribute Aβ accumulation (Silva et al., 2021). Several studies have reported AQP4 depolarization with AD pathology (Yang et al., 2011; Kress et al., 2014), notably in human post-mortem frontal cortex tissue (Zeppenfeld et al., 2017). Therefore, we conclude that AQP4 mislocalization not AQP4 deficiency could contribute to Aβ accumulation in the retina. Future studies of AQP4 and 6E10 colocalizations in the wholemounts of Ergo-treated and non-treated transgenic AD mouse models will confirm if the polarized AQP4 localization affects Aβ clearance process.

Finally, yet importantly, Chen et al.’s (2020) study in AD patients suggested that monocytes regulate blood levels of Aβ and might be involved in the development of AD. Approximately 40–60% of Aβ generated in the brain is estimated to diffuse into the blood and be cleared in the peripheral system (Qosa et al., 2014; Xiang et al., 2015; Yuede et al., 2016). Chen et al. (2020) suggest that monocytes might play a critical role in the clearance of brain derived Aβ in the periphery. On the other hand, OCTN1 mRNA expression data (Uhlén et al., 2015) shows that OCTN1 expressions are high in myeloid lineage cells such as granulocytes and monocytes. The functional expression of OCTN1 in intestinal immune cells and activated macrophages in ameliorating intestinal inflammation has been reported (Shimizu et al., 2015). Monocytes are capable of migrating into tissues where they transform into macrophages. In our work, colocalized 6E10 and IBA1 protein markers on the wholemounts strongly supported the presence of IBA1(+)ve macrophages that diffused from the blood vessels and engulfed the Aβ *via* phagocytosis (Figures 2G, 3E–H, Supplementary Images 1E, G, and Supplementary Videos 1, 2).

## 5. Conclusion

In this work, using the data from both planes of section (wholemount neuroretina vs. retinal cross-section), we explored the possible underlying mechanism of dietary antioxidant ergothioneine against Aβ accumulation in a transgenic 5XFAD mouse model. On the whole, despite the small sample size, significantly depleted astrocytes in 5XFAD mice compared with WT controls suggests lack of glymphatic drainage, a macroscopic waste clearance system mediated by astrocytic end-feet in the retinas of 5XFAD mice. Whereas improved Aβ clearance in Ergo-treated 5XFAD compared with non-treated 5XFAD supports our concept that Ergo stimulates Aβ clearance possibly by phagocytic IBA1(+)ve blood-derived macrophages and *via* perivascular drainage.

## Data availability statement

The original contributions presented in this study are included in the article/Supplementary material, further inquiries can be directed to the corresponding author.

## Ethics statement

This animal study was reviewed and approved by the Vanderbilt University’s Institutional Animal Care and Use Committee (IACUC) and The University of British Columbia Animal Care and Biosafety Committees.

## Author contributions

JM and WP generated the idea and obtained the funding. JM, WP, and PW proposed the hypothesis, designed the experiments, and interpreted the results. PW drafted the manuscript. PW, MC, CW, JH, and ET carried out the experiments. PW, MC, CL, and MT carried out the data analysis. All authors reviewed and approved the final manuscript.
