# Supplementary material for: Ergothioneine, a dietary antioxidant improves amyloid beta clearance in the neuroretina of a mouse model of Alzheimer’s disease
Source: Front Neurosci. 2023 Mar 14;17:1107436. doi: 10.3389/fnins.2023.1107436 (PMC10043244; doi:10.3389/fnins.2023.1107436)
Supplement: Supplementary file 1 [file Data_Sheet_1.docx]

**SUPPLEMENTARY MATERIALS**

**Image1:**Evidence for the existence of phagosome like structures (PLS) on the surface of wholemount neuroretinas. **(A)** Negative controlomitted for primary antibodies to distinguish debris or autofluorescence bodies (white circles); (**B,C,D**) GFAP+(ve) phagocytic astrocytes or PLS (red boxes) in WT controls, non-treated 5XFAD and Ergo-treated 5XFAD, respectively; (**E,F,G**) IBA1+(ve) phagocytic blood-derived macrophages or PLS (yellow boxes) in WT controls, non-treated 5XFAD and Ergo-treated 5XFAD, respectively. White circles in negative control show poor uptake of DAPI which distinguish the functional PLS from debris or autofluorescence bodies.(Scale bar 20µm; bright red – 6E10; bright green – IBA1/GFAP; blue – DAPI).

**Image 2:** Evidence for the existence of glymphatic and perivascular drainage system on the surface of wholemount neuroretinas.**(A)** Negative controlomitted for primary antibodies; 6E10 and GFAP colocalization revealed theexistence of glymphatic drainagein **(B)**Ergo-treated 5XFAD (white arrowheads) and (**C**) non-treated 5XFAD (white arrowheads), respectively.Aβ influx into the perivascular drainagevia capillary bed in (**E**) WT controls (yellow arrowheads) and (**F**)non-treated 5XFAD (yellow arrowheads). (Scale bars 20µm; bright red – 6E10; bright green – GFAP; blue – DAPI).

**Videos 1, 2:** Aβ clearance by infiltrated phagocytic IBA1(+)ve blood-derived macrophages identified on the surface of wholemount neuroretinas.

**Videos3, 4:** Aβ clearance by GFAP(+)vephagocytic astrocytes or PLS identified on the surface of wholemount neuroretinas.

**Videos5, 6:** Aβ clearance via glymphatic drainage identified on the surface of wholemount neuroretinas.

**Video7:** Aβ clearance by terminal end of the capillary bed identified on the surface of wholemount neuroretinas.

**SUPPLEMENTARY FILE**

For this work, we used 6E10 antibody (mouse monoclonal, Cat# 803001, BioLegend) to detect amyloid beta (Aβ) deposition and clearance in the wholemount neuroretinas and retinal cross-sections of ergothioneine (Ergo) treated 5XFAD, non-treated 5XFAD and non-treated C57BL/6J wildtype (WTcontrols). Antibody 6E10 immunoreactivity was detected in all three animal groups, although Aβ accumulation and clearance varied across them.

To date, no antibodies are available that properly discriminate endogenous rodent and transgenic human amyloid precursor protein (APP) in brains of APP-transgenic animals (1, 2). Holfling et al.’s (1) study clearly demonstrated that 6E10 is suitable to discriminate human and mouse APP only under reducing conditions in Western blot analysis but not in immunohistochemistry and immunocytochemistry. 6E10 detects background bands under nonreducing conditions which prevent a clear discrimination between wildtype and transgenic mice except for Tg2576 mice which have the highest APP expression. Nonreducing conditions in Western blots resemble the conditions during immunohistochemistry and immunocytochemistry more closely and are more indicative for the performance of antibodies in these applications (1, 2).

**Gene expression data:**

To support WT mice immunoreactivity to 6E10 antibody, we conducted a gene expression study in APP-PS1 double transgenic (Tg) mice, expressing a chimeric mouse/human *APP* (Mo/HuAPP695swe) and a mutant human presenilin 1 (*PSEN1*) (PS1-dE9), its non-carrier (NTg) siblings and WT controls to determine the relative level of human *APP* and *PSEN1* mRNAs in brain (neocortex-hippocampus) and eye tissues at young (3-4 months) and old (9-10 months) ages.

Our gene expression data in brain and eye tissues demonstrated a very strong level of expression of human *PSEN1* in Tg mice (*p*< 0.0001, fold change range 2x10^3^ – 1x10^6^), and almost same level of expression (*p*> 0.05) of human *APP* in Tg mice, NTg siblings and WT controls (**Image3**). On the other hand, our gene expression data in brain and eye tissues demonstrated a significant increase in mouse *App* mRNAlevels in Tg mice(*p*< 0.01, fold change range 2.1–3.7) compared with NTgsiblings or WT controls, andalmost same level of expression (*p*> 0.05) of mouse *Psen1* mRNA in Tg mice, NTgsiblings and WT controls (**Image4**).

*APP* and *PSEN1* mutations are associated with early-onset Alzheimer’s disease (AD). The amyloid hypothesis proposed that PSEN1 mutations initiate disease pathogenesis by increasing production of Aβ42 (3). PSEN1 functions as the catalytic subunit of γ-secretase, an intramembranous protease that cleaves APP. Following prior cleavage by β-secretase, processing of APP by γ-secretase generates Aβ peptides of varying lengths. Although Aβ40 accounts for ∼90% of Aβ production, the minor Aβ42 product is more hydrophobic and is thought to nucleate Aβ aggregation, leading to amyloid plaque deposition in the AD brain (3-5).  In sum,our gene expression data strongly supported the human PSEN1 mediated amyloid hypothesis in transgenic APP-PS1 mouse model.

In addition to above gene expression study, APP and PSEN1 protein canonical sequences obtained from UniProtKB (Swiss-Prot) database for human and mouse species were aligned and given below (**Sequence Alignments 1** and **2)**. It revealed 96.6% identity and 98.8% similarity between human and mouse APP protein canonical sequences, and 92.7% identity and 98.3% similarity between human and mouse PSEN1 protein canonical sequences.According to the manufacturer, the APP-PS1 double transgenic mouse expresses a "humanized" mouse amyloid beta (A4) precursor protein gene modified at three amino acids to reflect the human residues and further modified to contain the K670N/M671L mutations linked to familial AD (FAD). The PS1-dE9 transgene expresses a mutant human presenilin 1 carrying the exon-9-deleted variant (PSEN1dE9) associated with FAD. The 5XFAD transgenic mice overexpress mutant human amyloid beta (A4) precursor protein 695 (APP) with the Swedish (K670N, M671L), Florida (I716V), and London (V717I) familial AD mutations along with human presenilin 1 (PS1) harboring two FAD mutations, M146L and L286V.

On the whole, our gene expression study and sequence alignment data strongly supported the 6E10 immunoreactivity for human Aβ peptides in WT mice.

**SUPPLEMENTARY FIGURE LEGENDS**

**Image3:** Relative mRNA levels of human *APP* and *PSEN1* genes in transgenic APP-PS1 (Tg) mice, its non-carrier (NTg)siblings, and C57BL/6Jwildtype (WTcontrols) are illustrated. Total RNAs extracted from brain (neocortex-hippocampus) and eye tissues were used for cDNA preparation, followed by real-time quantitative reverse transcription PCR (RT-qPCR) with primers designed for human *APP* (accession ID NM_000484.4, forward- CACAGAGAGAACCACCAGCA and reverse- ACATCCGCCGTAAAAGAATG) and human *PSEN1* (accession ID NM_007318.3, forward- GGTAAAGCCTCAGCAACAGC and reverse- AAACAAGCCCAAAGGTGATG) genes. For data normalization, endogenous control glyceraldehyde-3-phosphate dehydrogenase (mouse *Gapdh*) was used. Gene expression levels were tested at 3-4 months (young) and at 9-10 months (old) ages. Cycle threshold (Ct) values obtained for *PSEN1* expression was very weak (≥ 35 cycles) in NTgsiblings and WT controls. Normalized Ct values (delta Ct) were compared between animal groups using Welch’s t*-*test at a significance level of *p*< 0.05 along with >2-fold intergroup difference to determine the differential expression. Human *APP* mRNAlevels were non-significant across the three animal groups (Tg, NTg and WT) at both young and old ages, and in both brain and eye tissues. Whereas human *PSEN1* mRNA level was abundantly and significantly high in Tg mice (*p*< 0.0001) at young and old ages, and in both brain and eye tissues.

**Image4:** Relative mRNA levels of mouse *App* and *Psen1* genes in transgenic APP-PS1 (Tg) mice, its non-carrier (NTg)siblings, and C57BL/6Jwildtype (WTcontrols) are illustrated. RT-qPCR was performed with primers designed for mouse *App* (accession ID NM_001198823.1, forward- GGTTCTGGGCTGACAAACAT and reverse- GTGATGACAATCACGGTTGC) and mouse *Psen1* (accession ID NM_001362271.1, forward- CGCACTACTGGACTGTGGAA and reverse- CAGTGCGGGTAAATCTCCAT) genes. For data normalization, endogenous control *Gapdh* was used. Gene expression levels were tested at 3-4 months (young) and at 9-10 months (old) ages. Delta Ct values were compared between animal groups using Welch’s t-test at a significance level of *p*< 0.05 along with >2-fold intergroup difference to determine the differential expression. Mouse *App* mRNA levels were significantly different(*p*< 0.01) across the three animal groups (Tg, NTg and WT) at both ages (young and old), and in both brain and eye tissues. Whereas mouse *Psen1* mRNA level was similar across the three animal groups atyoung and old ages, and in both brain and eye tissues.

**PAIRWISE SEQUENCE ALIGNMENT**

1) Amyloid precursor protein (APP)

>sp|P05067|A4_HUMAN Amyloid-beta precursor protein

>sp|P12023|A4_MOUSE Amyloid-beta precursor protein

Length: 770

Identity: 744/770 (96.6%)

Similarity: 761/770 (98.8%)

10 20 30 40 50 60

sp|P05 MLPGLALLLLAAWTARALEVPTDGNAGLLAEPQIAMFCGRLNMHMNVQNGKWDSDPSGTK

:::.::::::::::.::::::::::::::::::::::::.::::::::::::.:::::::

sp|P12 MLPSLALLLLAAWTVRALEVPTDGNAGLLAEPQIAMFCGKLNMHMNVQNGKWESDPSGTK

10 20 30 40 50 60

70 80 90 100 110 120

sp|P05 TCIDTKEGILQYCQEVYPELQITNVVEANQPVTIQNWCKRGRKQCKTHPHFVIPYRCLVG

::: :::::::::::::::::::::::::::::::::::::::::::: :.:::::::::

sp|P12 TCIGTKEGILQYCQEVYPELQITNVVEANQPVTIQNWCKRGRKQCKTHTHIVIPYRCLVG

70 80 90 100 110 120

130 140 150 160 170 180

sp|P05 EFVSDALLVPDKCKFLHQERMDVCETHLHWHTVAKETCSEKSTNLHDYGMLLPCGIDKFR

::::::::::::::::::::::::::::::::::::::::::::::::::::::::::::

sp|P12 EFVSDALLVPDKCKFLHQERMDVCETHLHWHTVAKETCSEKSTNLHDYGMLLPCGIDKFR

130 140 150 160 170 180

190 200 210 220 230 240

sp|P05 GVEFVCCPLAEESDNVDSADAEEDDSDVWWGGADTDYADGSEDKVVEVAEEEEVAEVEEE

::::::::::::::.:::::::::::::::::::::::::.::::::::::::::.::::

sp|P12 GVEFVCCPLAEESDSVDSADAEEDDSDVWWGGADTDYADGGEDKVVEVAEEEEVADVEEE

190 200 210 220 230 240

250 260 270 280 290 300

sp|P05 EADDDEDDEDGDEVEEEAEEPYEEATERTTSIATTTTTTTESVEEVVREVCSEQAETGPC

::::::: ::::::::::::::::::::::: ::::::::::::::::::::::::::::

sp|P12 EADDDEDVEDGDEVEEEAEEPYEEATERTTSTATTTTTTTESVEEVVREVCSEQAETGPC

250 260 270 280 290 300

310 320 330 340 350 360

sp|P05 RAMISRWYFDVTEGKCAPFFYGGCGGNRNNFDTEEYCMAVCGSAMSQSLLKTTQEPLARD

::::::::::::::::.::::::::::::::::::::::::::. .:::::::.::: .:

sp|P12 RAMISRWYFDVTEGKCVPFFYGGCGGNRNNFDTEEYCMAVCGSVSTQSLLKTTSEPLPQD

310 320 330 340 350 360

370 380 390 400 410 420

sp|P05 PVKLPTTAASTPDAVDKYLETPGDENEHAHFQKAKERLEAKHRERMSQVMREWEEAERQA

: ::::::::::::::::::::::::::::::::::::::::::::::::::::::::::

sp|P12 PDKLPTTAASTPDAVDKYLETPGDENEHAHFQKAKERLEAKHRERMSQVMREWEEAERQA

370 380 390 400 410 420

430 440 450 460 470 480

sp|P05 KNLPKADKKAVIQHFQEKVESLEQEAANERQQLVETHMARVEAMLNDRRRLALENYITAL

::::::::::::::::::::::::::::::::::::::::::::::::::::::::::::

sp|P12 KNLPKADKKAVIQHFQEKVESLEQEAANERQQLVETHMARVEAMLNDRRRLALENYITAL

430 440 450 460 470 480

490 500 510 520 530 540

sp|P05 QAVPPRPRHVFNMLKKYVRAEQKDRQHTLKHFEHVRMVDPKKAAQIRSQVMTHLRVIYER

:::::::.::::::::::::::::::::::::::::::::::::::::::::::::::::

sp|P12 QAVPPRPHHVFNMLKKYVRAEQKDRQHTLKHFEHVRMVDPKKAAQIRSQVMTHLRVIYER

490 500 510 520 530 540

550 560 570 580 590 600

sp|P05 MNQSLSLLYNVPAVAEEIQDEVDELLQKEQNYSDDVLANMISEPRISYGNDALMPSLTET

::::::::::::::::::::::::::::::::::::::::::::::::::::::::::::

sp|P12 MNQSLSLLYNVPAVAEEIQDEVDELLQKEQNYSDDVLANMISEPRISYGNDALMPSLTET

550 560 570 580 590 600

610 620 630 640 650 660

sp|P05 KTTVELLPVNGEFSLDDLQPWHSFGADSVPANTENEVEPVDARPAADRGLTTRPGSGLTN

:::::::::::::::::::::: ::.::::::::::::::::::::::::::::::::::

sp|P12 KTTVELLPVNGEFSLDDLQPWHPFGVDSVPANTENEVEPVDARPAADRGLTTRPGSGLTN

610 620 630 640 650 660

670 680 690 700 710 720

sp|P05 IKTEEISEVKMDAEFRHDSGYEVHHQKLVFFAEDVGSNKGAIIGLMVGGVVIATVIVITL

::::::::::::::: ::::.::.::::::::::::::::::::::::::::::::::::

sp|P12 IKTEEISEVKMDAEFGHDSGFEVRHQKLVFFAEDVGSNKGAIIGLMVGGVVIATVIVITL

670 680 690 700 710 720

730 740 750 760 770

sp|P05 VMLKKKQYTSIHHGVVEVDAAVTPEERHLSKMQQNGYENPTYKFFEQMQN

::::::::::::::::::::::::::::::::::::::::::::::::::

sp|P12 VMLKKKQYTSIHHGVVEVDAAVTPEERHLSKMQQNGYENPTYKFFEQMQN

730 740 750 760 770

1

42

16

672

713

687

Hu APP **IKTEEISEVKMDAEFRHDSGYEVHHQKLVFFAEDVGSNKGAIIGLMVGGVVIATVIVITL** 720

Mo APP **IKTEEISEVKMDAEFGHDSGFEVRHQKLVFFAEDVGSNKGAIIGLMVGGVVIATVIVITL** 720

Tg APP **IKTEEISEVNLDAEFRHDSGYEVHHQKLVFFAEDVGSNKGAIIGLMVGGVVIATVVIITL** 720

**SequenceAlignment 1:** Pairwise sequence alignment was conducted with GGSEARCH2SEQ that finds an optimal global alignment using the Needleman-Wunsch algorithm in Clustal Omega < Pairwise Sequence Alignment < EMBL-EBI Tool. It revealed 96.6% identity and 98.8% similarity between human and mouse APP protein canonical sequences. 1-42 amino acid residues of amyloid beta (Aβ) protein are highlighted in blue letters and separately shown in yellow highlights. Where only one amino acid pair arginine (R) and glycine (G) is mismatched (red color) between human and mouse species (R676G). Amino acid pairs tyrosine (Y) and phenylalanine (F), and histidine (H) and arginine (R) are functionally similar, categorized into same side chain groups (green color). Swedish (K670N, M671L), Florida (I716V), and London (V717I) familial AD mutations are indicated in blue color in transgenic mice APP. 6E10 antibody (Cat# 803001, BioLegend) is reactive to amino acid residues 1-16 and the epitope lies within amino acids 3-8 of Aβ peptide (EFRHDS).

2) Presenilin 1 (PSEN1)

>sp|P49768|PSN1_HUMAN Presenilin-1

>sp|P49769|PSN1_MOUSE Presenilin-1

Length: 467

Identity: 433/467 (92.7%)

Similarity: 459/467 (98.3%)

10 20 30 40 50 60

sp|P68 MTELPAPLSYFQNAQMSEDNHLSNTVRSQNDNRERQEHNDRRSLGHPEPLSNGRPQGNSR

:::.:::::::::::::::.: :...:::::..:::...::. : .:::.::::::.:::

sp|P69 MTEIPAPLSYFQNAQMSEDSHSSSAIRSQNDSQERQQQHDRQRLDNPEPISNGRPQSNSR

10 20 30 40 50 60

70 80 90 100 110 120

sp|P68 QVVEQDEEEDEELTLKYGAKHVIMLFVPVTLCMVVVVATIKSVSFYTRKDGQLIYTPFTE

::::::::::::::::::::::::::::::::::::::::::::::::::::::::::::

sp|P69 QVVEQDEEEDEELTLKYGAKHVIMLFVPVTLCMVVVVATIKSVSFYTRKDGQLIYTPFTE

70 80 90 100 110 120

130 140 150 160 170 180

sp|P68 DTETVGQRALHSILNAAIMISVIVV**M**TILLVVLYKYRCYKVIHAWLIISSLLLLFFFSFI

::::::::::::::::::::::::.:::::::::::::::::::::::::::::::::::

sp|P69 DTETVGQRALHSILNAAIMISVIVI**M**TILLVVLYKYRCYKVIHAWLIISSLLLLFFFSFI

130 140 **L** 150 160 170 180

190 200 210 220 230 240

sp|P68 YLGEVFKTYNVAVDYITVALLIWNFGVVGMISIHWKGPLRLQQAYLIMISALMALVFIKY

:::::::::::::::.:::::::::::::::.::::::::::::::::::::::::::::

sp|P69 YLGEVFKTYNVAVDYVTVALLIWNFGVVGMIAIHWKGPLRLQQAYLIMISALMALVFIKY

190 200 210 220 230 240

250 260 270 280 290 300

sp|P68 LPEWTAWLILAVISVYDLVAVLCPKGPLRMLVETAQERNETLFPA**L**IYSSTMVWLVNMAE

::::::::::::::::::::::::::::::::::::::::::::::::::::::::::::

sp|P69 LPEWTAWLILAVISVYDLVAVLCPKGPLRMLVETAQERNETLFPA**L**IYSSTMVWLVNMAE

250 260 270 280 **V** 290 300

310 320 330 340 350 360

sp|P68 GDPEAQRRVSKNSKYNAESTERESQDTVAENDDGGFSEEWEAQRDSHLGPHRSTPESRAA

::::::::: :: :::.. .:::.::. . ::::::::::::::::::::::::::::::

sp|P69 GDPEAQRRVPKNPKYNTQRAERETQDSGSGNDDGGFSEEWEAQRDSHLGPHRSTPESRAA

310 320 330 340 350 360

370 380 390 400 410 420

sp|P68 VQELSSSILAGEDPEERGVKLGLGDFIFYSVLVGKASATASGDWNTTIACFVAILIGLCL

:::::.:::..:::::::::::::::::::::::::::::::::::::::::::::::::

sp|P69 VQELSGSILTSEDPEERGVKLGLGDFIFYSVLVGKASATASGDWNTTIACFVAILIGLCL

370 380 390 400 410 420

430 440 450 460

sp|P68 TLLLLAIFKKALPALPISITFGLVFYFATDYLVQPFMDQLAFHQFYI

:::::::::::::::::::::::::::::::::::::::::::::::

sp|P69 TLLLLAIFKKALPALPISITFGLVFYFATDYLVQPFMDQLAFHQFYI

430 440 450 460

**SequenceAlignment 2:** Pairwise sequence alignment was conducted with GGSEARCH2SEQ that finds an optimal global alignment using the Needleman-Wunsch algorithm in Clustal Omega < Pairwise Sequence Alignment < EMBL-EBI Tool. It revealed 92.7% identity and 98.3% similarity between human and mouse PSEN1 protein canonical sequences. 5XFAD mice overexpressing human PSEN1 harboring two FAD mutations, M146L and L286V are indicated in red letters.

**REFERENCES**

1)Höfling C, Morawski M, Zeitschel U, Zanier ER, Moschke K, Serdaroglu A, et al. Differential transgene expression patterns in Alzheimer mouse models revealed by novel human amyloid precursor protein-specific antibodies. *Aging Cell* (2016) 15:953-63. doi: 10.1111/acel.12508

2) Sidiqi A, Wahl D, Lee S, Ma D, To E, Cui J, et al. *In vivo* Retinal Fluorescence Imaging With Curcumin in an Alzheimer Mouse Model. *Front Neurosci.* (2020) 14:713. doi: 10.3389/fnins.2020.00713

3) Hardy J, Selkoe DJ. The amyloid hypothesis of Alzheimer's disease: progress and problems on the road to therapeutics. *Science* (2002) 297:353-6. doi: 10.1126/science.1072994. Erratum in: Science (2002) 297:2209.

4) Duff K, Eckman C, Zehr C, Yu X, Prada CM, Perez-tur J, et al. Increased amyloid-beta42(43) in brains of mice expressing mutant presenilin 1. *Nature* (1996) 383:710-3. doi: 10.1038/383710a0

5) Borchelt DR, Thinakaran G, Eckman CB, Lee MK, Davenport F, Ratovitsky T, et al. Familial Alzheimer's disease-linked presenilin 1 variants elevate Abeta1-42/1-40 ratio in vitro and in vivo. *Neuron* (1996) 17:1005-13. doi: 10.1016/s0896-6273(00)80230-5
