# Supplementary figures and images for: Ergothioneine, a dietary antioxidant improves amyloid beta clearance in the neuroretina of a mouse model of Alzheimer’s disease
Source: Front Neurosci. 2023 Mar 14;17:1107436. doi: 10.3389/fnins.2023.1107436 (PMC10043244; doi:10.3389/fnins.2023.1107436)

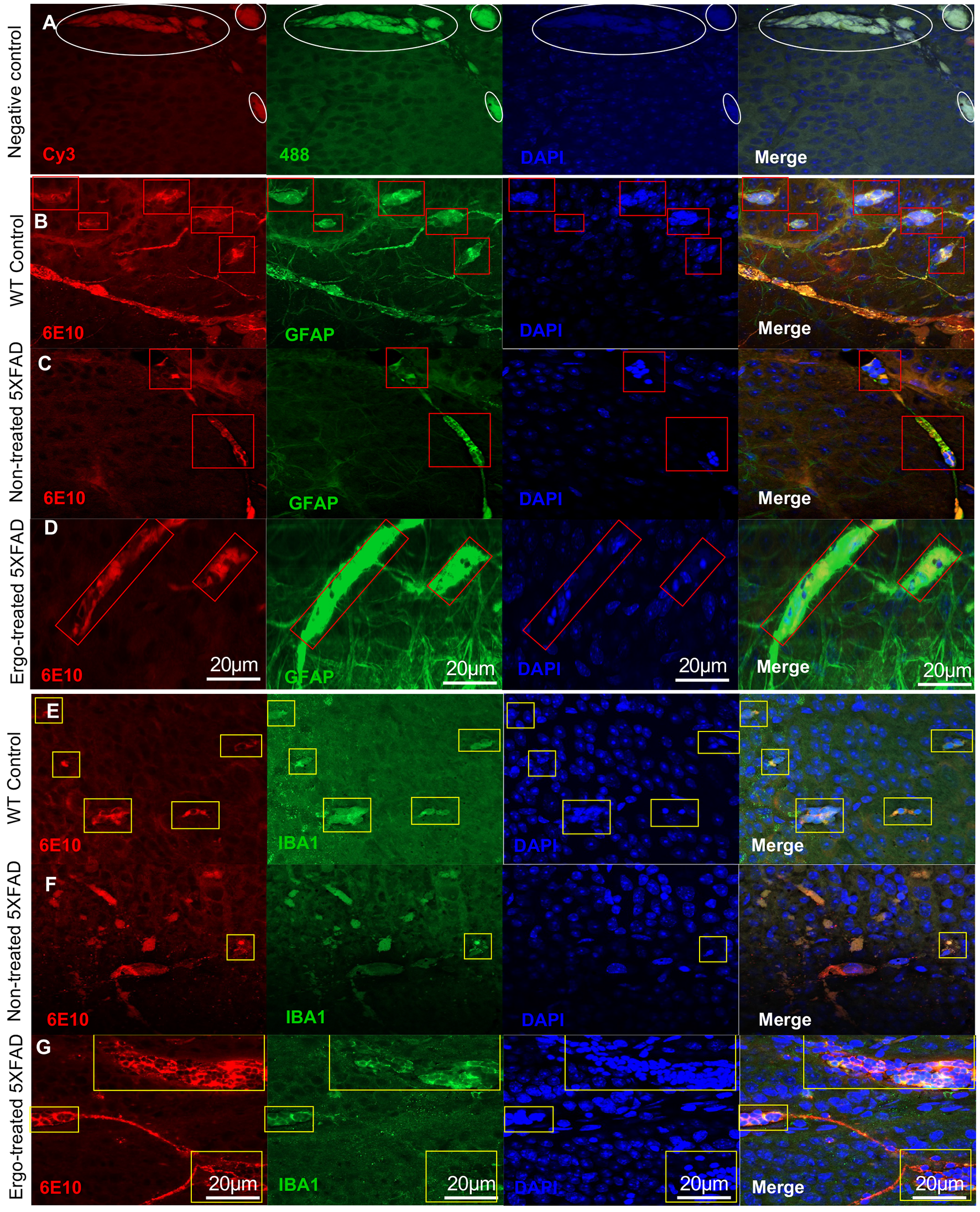

Supplement: Supplementary file 9 [file Image_1.TIF]

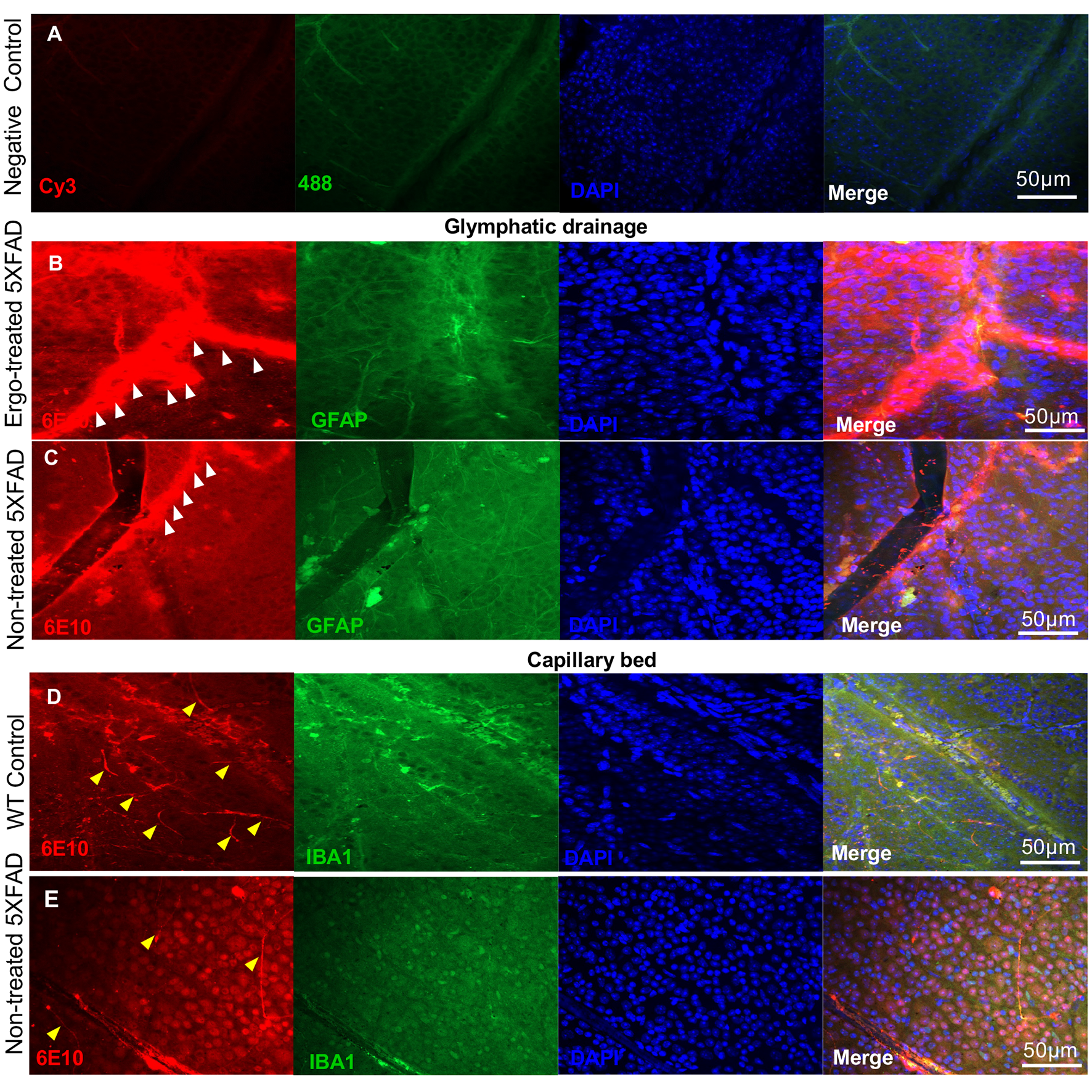

Supplement: Supplementary file 10 [file Image_2.TIF]

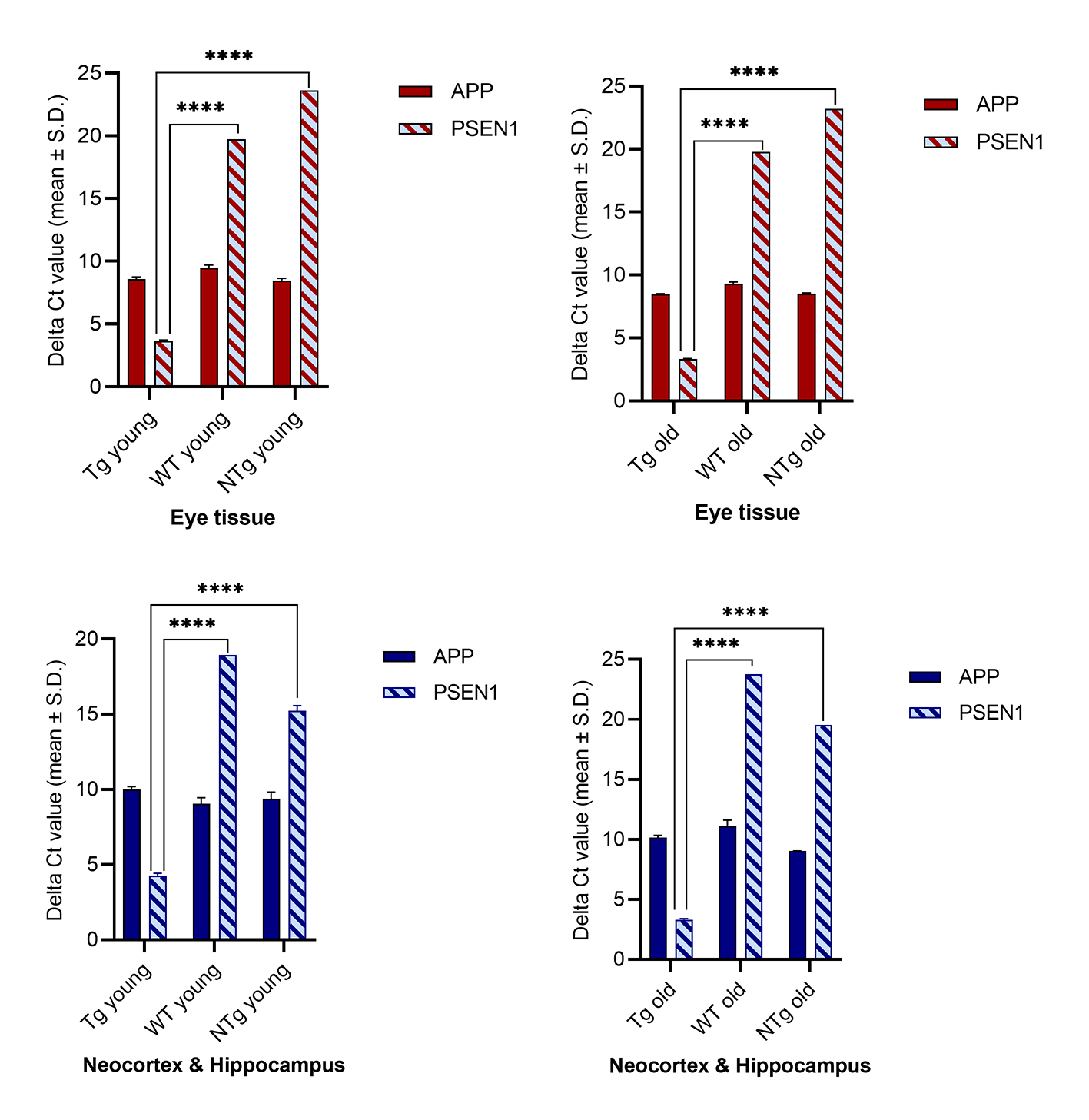

Supplement: Supplementary file 11 [file Image_3.TIF]

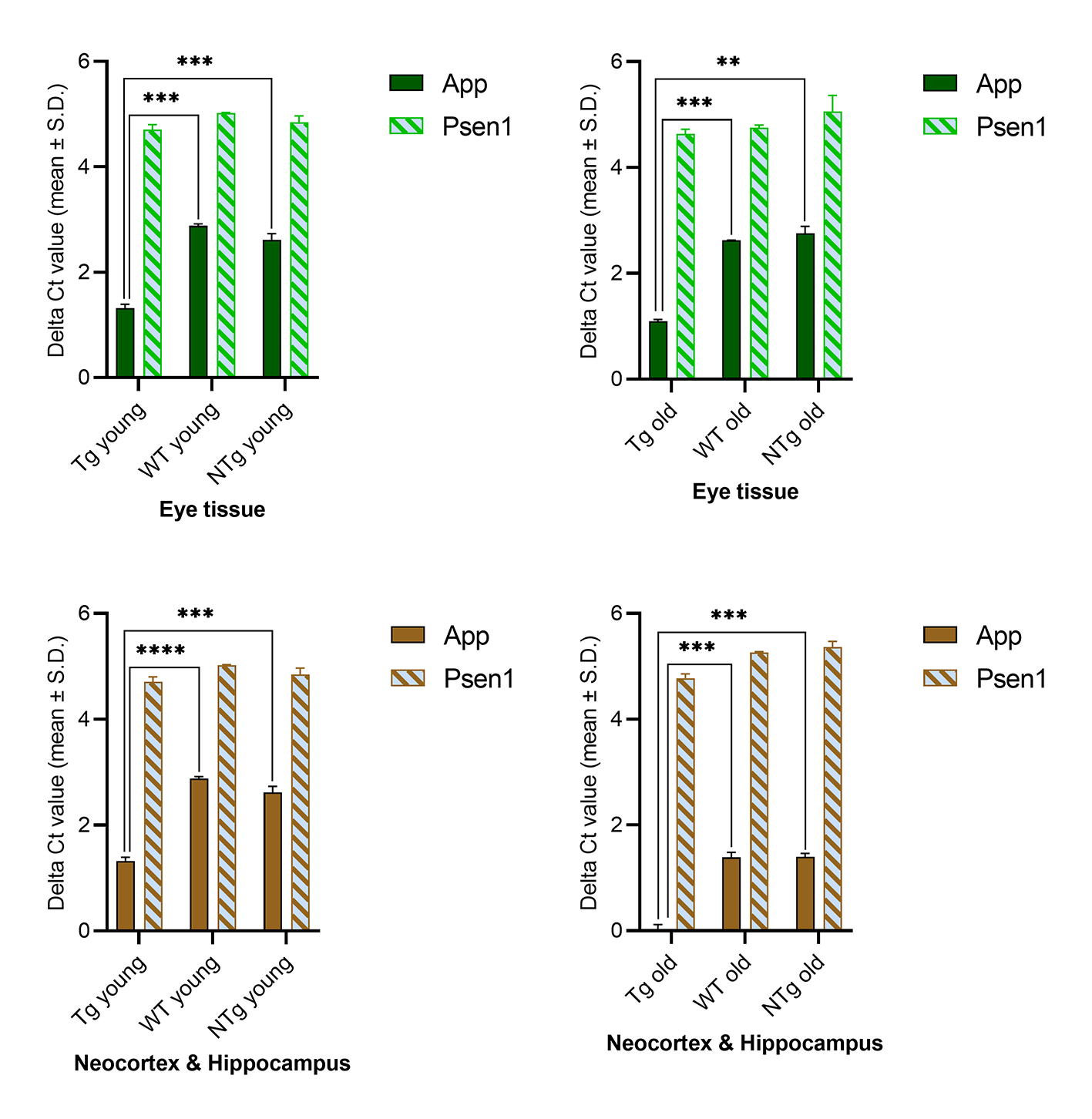

Supplement: Supplementary file 12 [file Image_4.TIF]
